# Identification of Design Principles for the Preparation of Colloidal Plexcitonic Materials

**DOI:** 10.1021/acs.langmuir.3c01642

**Published:** 2023-08-29

**Authors:** Nicola Peruffo, Matteo Bruschi, Barbara Fresch, Fabrizio Mancin, Elisabetta Collini

**Affiliations:** † Department of Chemical Sciences, University of Padova, via Marzolo 1, 35131 Padova, Italy; ‡ Padua Quantum Technologies Research Center, via Gradenigo 6/A, 35122 Padova, Italy

## Abstract

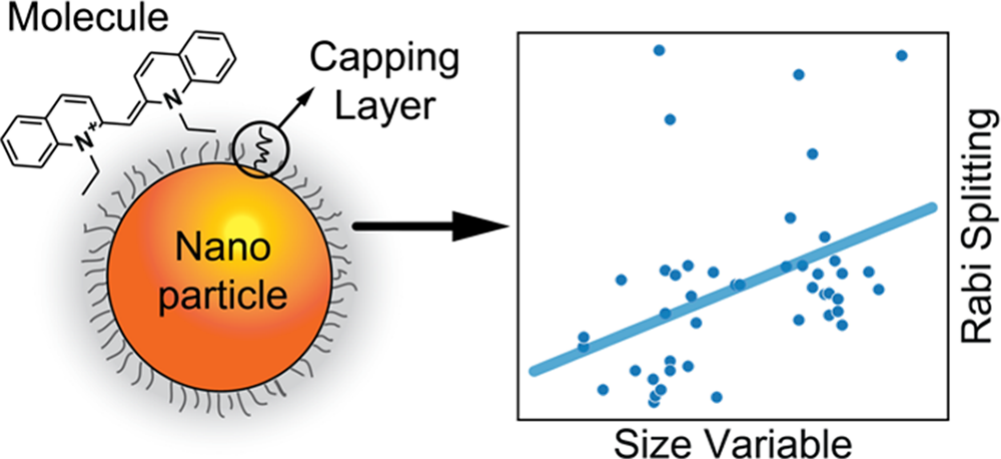

Colloidal plexcitonic materials (CPMs) are a class of nanosystems where molecular dyes are strongly coupled with colloidal plasmonic nanoparticles, acting as nanocavities that enhance the light field. As a result of this strong coupling, new hybrid states are formed, called plexcitons, belonging to the broader family of polaritons. With respect to other families of polaritonic materials, CPMs are cheap and easy to prepare through wet chemistry methodologies. Still, clear structure-to-properties relationships are not available, and precise rules to drive the materials’ design to obtain the desired optical properties are still missing. To fill this gap, in this article, we prepared a dataset with all CPMs reported in the literature, rationalizing their design by focusing on their three main relevant components (the plasmonic nanoparticles, the molecular dyes, and the capping layers) and identifying the most used and efficient combinations. With the help of statistical analysis, we also found valuable correlations between structure, coupling regime, and optical properties. The results of this analysis are expected to be relevant for the rational design of new CPMs with controllable and predictable photophysical properties to be exploited in a vast range of technological fields.

## Introduction

**Figure 1:**
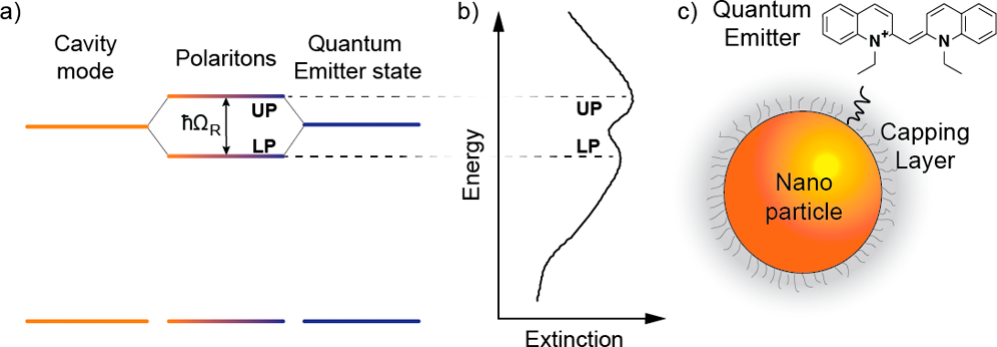
(a) Level diagram of a coupled QE–cavity system showing the formation of new hybrid polaritonic states UP and LP. (b) Extinction spectrum of a polaritonic system where UP and LP are identified. (c) Schematization of a CPM system, where the three main constituents are highlighted: the plasmonic nanoparticle (NP), the molecular quantum emitters (QEs) adsorbed on the NP, and the NP capping layer (CL).

The coupling of light and matter, even at the nanometric scale, has been long investigated using molecular quantum emitters (QEs) in “cavities”, i.e., structures where the electromagnetic field of the light is amplified.^1−3^ The light–matter coupling can be classified as weak or strong, depending on the relative ratio between the rate of the energy exchange among the QE and cavity components and the respective dissipation rates. In the weak coupling regime, the dissipation processes are faster than the energy exchange: the QEs and the cavity maintain most of their individual properties, but QEs’ emission lifetime can be suppressed or enhanced as a consequence of the Purcell effect.^4−6^ In the strong coupling (SC) regime, instead, the back-and-forth coherent energy exchange between the QEs and the cavity predominates over dissipation.^1^ This, in turn, produces two bright hybrid light–matter states, called upper and lower polaritons (UP and LP, Figure 1a,b).^1,7^ The frequency gap between these states is called Rabi splitting (Ω_R_), and it corresponds to the frequency of the energy exchange.^7,8^

Polaritonic materials are currently attracting considerable interest in several fields. For example, regarding chemistry applications, it was demonstrated that these polaritonic systems could be effectively employed to tune the rate of chemical reactions,^9,10^ increasing the efficiency of molecular energy transfer,^11,12^ or modifying the temporal and spatial propagation of the energy.^13−15^ In addition, cavities small enough [such as plasmonic nanoparticles (NPs) or arrays] can confine the light below the diffraction limits, potentially unlocking an unprecedented capability of directing the migration of the excitation energy at the nanoscale.^16,17^ They have also been proposed as suitable candidates for sensing,^18^ imaging,^19,20^ and for different kinds of devices such as parametric amplifiers,^21^ OLED,^22^ and solar cells.^22−26^ More recently, several efforts were also paid to characterize and exploit their quantum properties, such as superfluidity,^27,28^ superconductivity,^27,29^ Bose–Einstein condensation,^30−32^ and coherent behavior.^33,34^

Polaritonic materials are essentially prepared with two classes of cavities: the Fabry–Pérot (or planar) cavities and plasmonic cavities. In the first class, the light bounces back and forth between two parallel mirrors forming standing waves, whose fields are amplified.^3,35^ In the second class, the field is enhanced by the plasmonic resonances that can be localized on single plasmonic NPs or delocalized along a patterned structure, as in the case of plasmonic arrays (Table 1).^36,37^

**Table 1:** Different Cavity Systems Used To Establish Light–Matter Coupling and Their Main Features

	Fabry–Pérot cavities	plasmonic arrays	colloidal plasmonic NPs
effective volume	high	low	low
losses	low	low/high	high
diffraction limit	above	below	below
most common preparation methods	sputtering, evaporation, chemical vapor deposition	sputtering, evaporation, electrodeposition, nanolithography	wet chemistry

The polaritonic states formed when plasmonic cavities are coupled with excitonic QEs are more specifically called plexcitons. The study of plexcitonic materials built with plasmonic arrays is reviewed in several studies.^1,3,5,38^ Recently, colloidal NPs have been reported as suitable cavities (Figure 1c). Plasmonic cavities confine the light field below the diffraction limit in a small volume, allowing the control of the light flow at the nanoscale level, increasing their potential application impact in the photonic field and for all-optical devices. In such a small volume, called “effective volume” *V*_eff_, the electromagnetic field is extraordinarily enhanced, favoring the establishment of an SC regime, even if the dissipative rates are generally higher than in Fabry–Pérot cavities, as explained in the next section. Moreover, unlike Fabry–Pérot cavities and plasmonic arrays, usually prepared by expensive physical methods (Table 1), colloidal NPs are easy and cheap to synthesize by simple wet chemistry methods.

**Figure 2:**
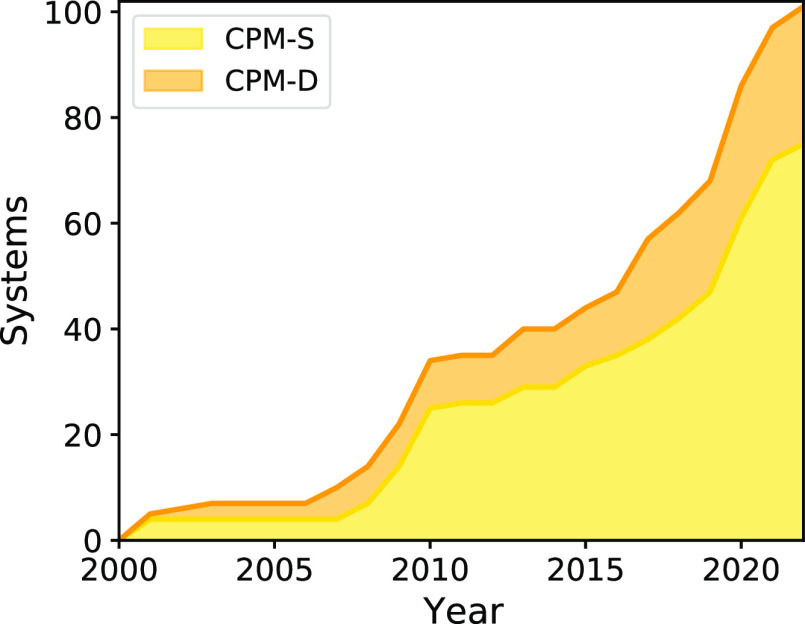
Timeline of the study of CPMs. CPMs are classified into systems dispersed in solution (CPM-S, yellow area) and deposited onto a solid support (CPM-D, orange area).

This article focuses precisely on the so-called “colloidal plexcitonic materials” (CPMs), prepared using colloidal NPs as plasmon cavities. The interest in this kind of plexcitonic materials is relatively recent, and their photophysical properties are still poorly explored. To the best of our knowledge, the first papers on this subject were published in 2001,^39,40^ and the overall literature consists of about 70 papers and 100 systems that are studied so far (Figure 2). Clear structure-to-properties relationships are not available yet, and thus a survey of the systems presented in the literature and their coupling properties appears timely. Therefore, in this work, we collect in a dataset all the CPMs so far reported, to our best knowledge, with the aim of rationalizing their design and providing valuable guidelines for the preparation of novel nanohybrids with controlled and predictable properties. In general, the preparation of a CPM requires conjugating a plasmonic NP and an organic dye with similar absorption maxima. Once the two components are selected, the main issue is to find out a strategy to induce the conjugations (covalent binding, adsorption, and deposition). Consequently, for the first categorization, the CPMs have been classified according to their three main constituents: (i) the plasmonic NP, (ii) the molecular QEs adsorbed on the NP, and (iii) the NP capping layer (CL), which stabilizes the NPs but also plays the important role of modulating the interactions between the NP and the QEs (Figure 1c).^41,42^ Additionally, as a further evaluation criterion, we distinguish between CPMs dispersed in solution (CPM-S) and those deposited onto a solid-state substrate (CPM-D). Once the most common designs reported in the literature are identified, our analysis continues by assessing statistically significant correlations between selected structures and the optical properties in the plexcitonic systems prepared and analyzed to date. To this aim, we adopt a statistical approach based on a physically sensible definition of the (dependent) response variable and of the (independent) predictor variables, in this case, the physicochemical characterization of CPMs in terms of their structural components.

## Materials and Methods

### Quantum Model of Plexciton Formation

A key concept in the description of the light–matter coupling is that the strength of the coupling depends on the transition dipole moment of the QE (μ) and the electromagnetic field enhanced by the cavity mode (ξ). The corresponding matrix element of the coupling Hamiltonian *H*_int_ in the Jaynes–Cummings model can be written as^5^
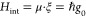
1where *ℏ* is the reduced Planck constant and *g*_0_ is the coupling strength between the single QE and the field. To identify different coupling regimes, *g*_0_ is usually compared with the decay rates (or losses) of the cavity and the QE, indicated as *k* and γ, respectively. Indeed, when *g*_0_*> k,* γ, the system can enter the SC regime, where polaritonic states arise.

Since most of the cavities are large enough to host more than one QE, polaritonic states are usually produced by the coupling of many QEs to the same cavity. This situation is most commonly described by using the Dicke or Tavis–Cumming model. This approach is a fully quantum mechanical model that treats a collection of *N* QEs as a giant quantum oscillator.^7,43−45^ According to this model, the Hamiltonian is^46^
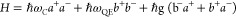
2where *a*^+^ and *a*^–^ are the creation and annihilation operators of the electromagnetic mode; *b*^+^ and *b*^–^ are the creation and annihilation operators for the QE giant oscillator;^47^ and *ℏ*ω_C_ and *ℏ*ω_QE_ are the energies of the cavity mode and the QE, respectively. The third term of the Hamiltonian is *H*_int_, where the overall coupling strength *g* is related to the coupling strength *g*_0_ of the individual QEs according to the relation *g* = *N*^1/2^*g*_0_. Hence, the number *N* of QEs plays a key role in the overall coupling of the system. Assuming a resonant interaction, where the detuning δ = *ℏ*ω_C_ – *ℏ*ω_QE_ = 0, two new bright eigenstates (UP and LP) are formed:^48^
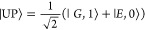
3a
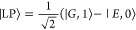
3bwhere |*G*,1⟩ is the state in which the QEs are in the collective ground state |*G*⟩ and the cavity mode is excited |1⟩; conversely, |*E*,0⟩ is the state in which the QEs are in the collective excited state |*E*⟩ and the cavity mode is in the vacuum state |0⟩. The eigenfrequencies corresponding with these two states are
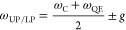
4

In resonance conditions, the new polaritonic eigenstates are equally composed of the cavity and the QEs, and thus it is no longer possible to treat the cavity and the QEs independently. In addition to UP and LP, there are other *N* – 1 states which form a dark manifold of states due to symmetry considerations.^48^

Equation 4 does not account for the coherence dephasing occurring because of the dissipation rates (or losses) of the QE and the plasmonic cavity. The dephasing can be considered by introducing phenomenologically the decay rates as complex frequencies for the QE: ω_QE_ – *i*γ, and the cavity: ω_C_ – *ik*. The resulting eigenfrequencies, found by diagonalizing a non-Hermitian Hamiltonian, become complex^1^
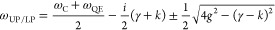
5and the Rabi splitting Ω_R_:
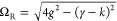
6Ω_R_ is thus dependent on the decay rates that consequently reduce the overall splitting between UP and LP.

As discussed earlier, an important parameter controlling the coupling is the electric field vacuum fluctuations strength ξ^7^ (eq 2). For a cavity, it can be calculated as .^50^*V*_eff_ is the effective volume, i.e., the volume where the field of the light is enhanced by the cavity. By introducing this equation in the coupling Hamiltonian of many QEs coupled with a cavity, we obtain
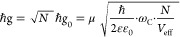
7

This expression explains why CPMs, and in general polaritonic materials prepared with plasmonic cavities, can often generate a Rabi splitting in the same order of magnitude or greater than Fabry–Pérot cavities despite their huge losses. The plasmonic effective volume, indeed, is orders of magnitude smaller than in Fabry–Pérot cavities.^5,6^ However, the effective volume described as such does not consider the plasmonic losses, which cannot be neglected. For this reason, it is recognized that this definition is not completely accurate for plasmonic cavities, and nowadays, there is still an open and vibrant debate about possible more reliable definitions.^5^

The nanosystems where the coupling strength *g* overcomes the dissipation rates γ and *k* enter the SC regime. To define this regime, several studies refer to the generic relation 2*g > |k* – γ*|*, which considers that the square root argument on the right side of eq 6 must be positive.^7,8,49^ This is equivalent to the condition Ω_R_ > 0. However, the condition Ω_R_ > 0 is verified in plasmon-exciton systems even without the effective formation of polaritonic states. Indeed, between the weak coupling and the strong one, there is a third regime that can be defined as “intermediate”.^50,51^ In this regime, the electromagnetic fields of the plasmon and the QEs can interfere destructively, giving rise to a Fano resonance, producing a dip in the extinction spectrum in correspondence with the molecular transition.^52−54^ Distinguishing the Fano interference-type behavior from the Rabi splitting-type behavior expected upon plexciton formation is a challenging task because there are not net boundaries between the two regimes.^51,54,55^ Moreover, Stete et al. demonstrated that the typical plexcitonic splitting in the extinction spectrum could occur even before the SC regime is established because the vacuum fluctuations can saturate the QE optical transition.^56^ For this reason, the electronic states of some nanohybrids were called Fano plexcitons.^23,57^ In this framework, it is clear that the inequality 2*g > |k* – γ*|* (or Ω_R_ > 0) cannot be used to identify univocally the formation of plexcitonic states. A stricter condition is that Ω_R_ frequency must be bigger than the dissipation rates of the uncoupled system:^58^
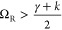
8

This condition guarantees, at least in principle, that QEs and cavities exchange the energy faster than dissipating it individually, producing new plexcitonic eigenstates. It is crucial to distinguish the SC regime from the intermediate regime because, although the extinction spectra show similar features in the two cases, the associated photophysical and dynamical behaviors are expected to be utterly different.

Note that eq 8 identifies the ideal SC condition and does not account for the complexity of the real samples, typically characterized by inhomogeneous distributions of size and shape of the NPs, and inhomogeneous distributions of distances and orientations of the molecular QEs onto the NPs surfaces. Notwithstanding these limitations, the SC condition in eq 8 can still be used as a rule of thumb to roughly distinguish the two different regimes.

Experimentally, the main parameters needed to quantify the coupling regime in CPMs (Ω_R_, *ℏ*γ, and *ℏk*) can be estimated by simply measuring the extinction spectra of the samples. Indeed, when plexcitons are formed, the plasmonic resonance in the extinction spectrum splits into two plexcitonic resonances, allowing for the quantification of *ℏ*Ω_R_ as the energy gap between them. *ℏ*γ and *ℏk*, instead, can be measured as the full width at half-maximum of the respective uncoupled plasmonic and excitonic peaks. It is worth noticing that CPMs, being prepared by wet chemistry methodologies, are intrinsically inhomogeneous samples, with distributions of NPs’ size and shape, thickness of the capping layer, number of coupled QEs, and so forth, which might slightly complicate the estimate of the Rabi splitting and coupling regime.^34^

### Plexciton Dataset

Even with the uncertainties related to the boundaries of the SC regime, the theoretical model discussed in the previous section clearly points out the main parameters controlling the coupling strength *g* and the Rabi splitting Ω_R_. These include the values of the QE transition dipole moment μ, the number *N* of QEs coupled with the cavity, the effective volume *V*_eff_, the frequency of the cavity ω_C_, and the decay rates γ and *k*. However, relating such parameters to specific properties of the main components of CPMs, i.e., NP, QEs, and CL (Figure 1c) is not straightforward. To do that, we built and statistically analyzed a dataset of all the colloidal plexcitonic nanosystems reported in the literature (see Section S1).

The dataset classifies each plexcitonic nanosystem according to different features, namely, the material and the shape of the NPs; the molecular structure, the family, and the aggregation state of the QE molecules; the CL structures and classes, and the nature of the supramolecular interaction between NPs and QEs. CPM-S and CPM-D are also distinguished. For each system, we report the value of *ℏ*γ, *ℏk*, and *ℏ*Ω_R_, the last one representing the energy gap between the plexcitonic states and calculated as the energy difference between the plexcitonic peaks in the extinction spectrum. The coupling regime can be evaluated using the SC condition reported in eq 8, which can be conveniently rearranged as 2Ω_R_/(γ + *k*) > 1. For brevity, from now on, we will introduce the Coupling Ratio, defined as CR≡2Ω_R_/(γ + *k*). CR is an intuitive way to indicate whether the system is in the SC regime just by evaluating if CR is above or below 1. The CR value is calculated for all the systems in our dataset as well. The surface area *S* of the NPs, their volume *V*, and the ratio *S/V* are also identified. The data are retrieved from the original publications or, when not explicitly reported, are calculated from the data available (see Section S3 for further explanation). The dataset comprises 101 samples, 75 in solution (CPM-S) and 26 deposited onto a solid substrate (CPM-D).

### Multiple Linear Regression Model

We analyze the dataset in terms of regression models to extract significant correlations between the plexcitonic character of the materials and their design components. Such statistical correlations emerge from a heterogeneous pool of plexcitonic systems that cannot be described by a common microscopic model. In this sense, the analysis circumvents the typical assumptions of the quantum-mechanical model introduced before, such as the simplification of the plasmonic response, regular and homogeneous NPs’ geometries, identical emitters uniformly distributed on the surface, and constant coupling strengths. To identify the relevant variables for the statistical models, we focus on the role of NPs, QEs, and CL in determining the SC condition.

The numerical variables available from the CPMs’ literature survey include the surface area *S* and volume *V* of the NP, the *ℏ*Ω_R_ and CR values, and the linewidths *ℏk* and *ℏ*γ estimating the decay rate of the plasmon and the QEs, respectively. On the other hand, all the other properties are qualitative, and therefore, they are categorical variables assuming one of a limited number of possible values. As an example, the NP material is a variable taking three possible values (Au, Ag, and Ag/Au). The other qualitative variables are the shape of the NPs (cubes, stars, bipyramids, shells, spheres, rods, hollow prisms, flat dodecahedra, disks, platelets, and prisms), the chemical nature of the CL (weak, polymers, surfactant, and thiols), the type of dye molecules (grouped into PIC, TDBC, DBTC, JC1, and others), and the interactions between dye and NP (classified as weak interactions and direct adsorption). To include these qualities into the regression model, we use a dummy encoding, where each of their possible values, like “being an Ag NP”, became a binary variable *D_j_* [0 (false) or 1 (true)] (see Section S4 for further details).

We chose a linear statistical model for estimating the value of one response variable *y* given a set of continuous (*x_j_*) and binary variables, *D_i_*:
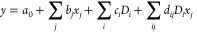
9

As the response variables *y*, we consider the energy associated with the Rabi splitting, *ℏ*Ω_R,_ and the coupling ratio, CR. Although the two variables are proportional to each other, they highlight different aspects of the coupling strength, as discussed below. The predicting variables are selected in different combinations; however, a maximum of three predicting variables can be reasonably included in one model due to the limited size and wide distribution of the dataset. The coefficients of the model in eq 9 are optimized simultaneously to best fit the dataset. In particular, *a*_0_ is the so-called reference state and represents the intercept of the model. The coefficients *b_j_* are the rate of change of the response variable *y* with the continuous variable *x_j_*. The coefficients of the third term *c_j_* introduce a different intercept of the linear relation if the variable *D_i_* is equal to 1 (true). The fourth term is the so-called “interaction term”, and it accounts for situations in which the simultaneous influence of the two variables *x_j_* and *D_i_* on the response is not additive, modifying the slope of the model.

The significance of each model is quantified by specific statistical metrics. In particular, the *R*^2^ parameter reflects the portion of the sample variance explained by the model, while the *p*-value associated with each coefficient assesses its statistical significance. We will discuss only regression models featuring *p*-values smaller than 0.05 for all the coefficients, meaning that the probability of highlighting a correlation that is not present is less than 5%. More technical details on the statistical models and their diagnostics can be found in Section S4. In the following, we highlight the significant correlations extracted from our dataset.

Not all the nanomaterials collected in the dataset could be included in this statistical analysis. In some cases, *ℏ*Ω_R_ could not be determined because of the low association constant, high detuning values (like in the case of nanohybrids prepared with TC dyes), or the presence of multiple couplings; in some others, *V* could not be estimated appropriately because no TEM images of the NPs were reported (see Section S3 for further information). On the other hand, when the same kinds of nanohybrids are prepared using NPs with different dimensions (see, for instance, refs (88) and (89)), all these samples are included independently to increase the statistics. Overall, the dataset scrutinized in the statistical analysis includes 71 CPMs (47 CPM-S and 24 CPM-D).

## Results and Discussion

### Distribution of *ℏ*Ω_R_ and Coupling Ratio

**Figure 3:**
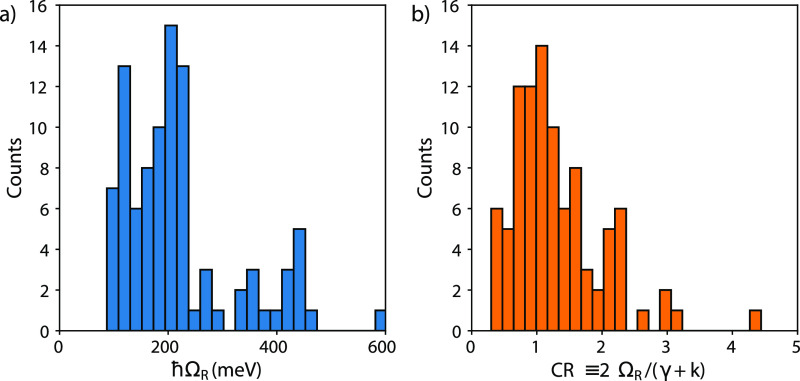
Histograms reporting the distributions of (a) Rabi splitting and (b) coupling ratio (CR) for the analyzed CPMs.

The histogram in Figure 3a reports the *ℏ*Ω_R_ distribution for the subset of CPMs where *ℏ*Ω_R_ could be measured (see Section S3 for further information). The *ℏ*Ω_R_ spreads between 87 and 603 meV, with 50% of the population distributed between 125 and 250 meV, values typically classified as belonging to the SC regime. These are typical values reported in the literature also for SC materials prepared with different kinds of cavities, such as Fabry–Pérot cavities, plasmonic arrays, or plasmonic dimers.^1,59^

In Figure 3b, the distribution of CR is reported. Noticeably, 38% of the CPMs fall below the SC conditions (CR < 1). Hence, in almost half of the cases, SC plexcitons are not obtained. This observation suggests that the requirements to achieve SC are still poorly understood, and that in most cases, a trial-and-error approach was likely used to guide the selection of the components of the plexcitonic hybrids. In the following sections, we will analyze the systems reported in the dataset to evaluate their main structural features and identify possible structure-to-properties relationships more clearly. Subsequently, these structural features will be related to the *ℏ*Ω_R_ and SC conditions through a detailed statistical regression model.

### Plasmonic NPs: Materials and Shapes

In Table 2, the CPMs are classified based on the metal and shape of the plasmonic NPs. All the CPMs reported so far are made with the most common plasmonic materials: Au, Ag, and Ag/Au alloys. Au is the predominantly adopted metal for CPM-S, while in the case of CPM-D, Au and Ag are equally used. The shapes of the plasmonic NPs are much more variable, spreading from the simplest geometries, such as rods and spheres, to the less common ones, such as rings or bipyramids. Spheres and rods are present in more than 60% of the cases, likely because they are relatively easy to prepare and well-known in the literature. Beyond them, stars and hollow prisms are the most used shapes in the case of CPM-S, while cubes and disk-like shapes (disks, flat dodecahedra, and platelets) are preferred in the case of CPM-D.

**Table 2:** Classification of the CPMs Based on the Metal and Shape of the Plasmonic Moieties^a^

plasmonic NPs			
material	CPM-S (%)	CPM-D (%)	CPM-S + CPM-D (%)
Ag	13	42	21
Ag/Au	22	15	21
Au	65	42	59

shape	CPM-S (%)	CPM-D (%)	CPM-S + CPM-D (%)
bipyramids	3	0	2
cubes	5	12	7
disk-like	8	8	8
hollow prisms	9	4	8
rods	34	23	31
shells	6	0	5
spheres	22	54	30
stars	13	0	10

a The percentages of samples prepared with different NP materials and shapes are reported. The first two columns distinguish CPM-S and CPM-D, while the cumulative data are shown in the last column.

### Dyes: Molecular Structures and Aggregation States

**Figure 4:**
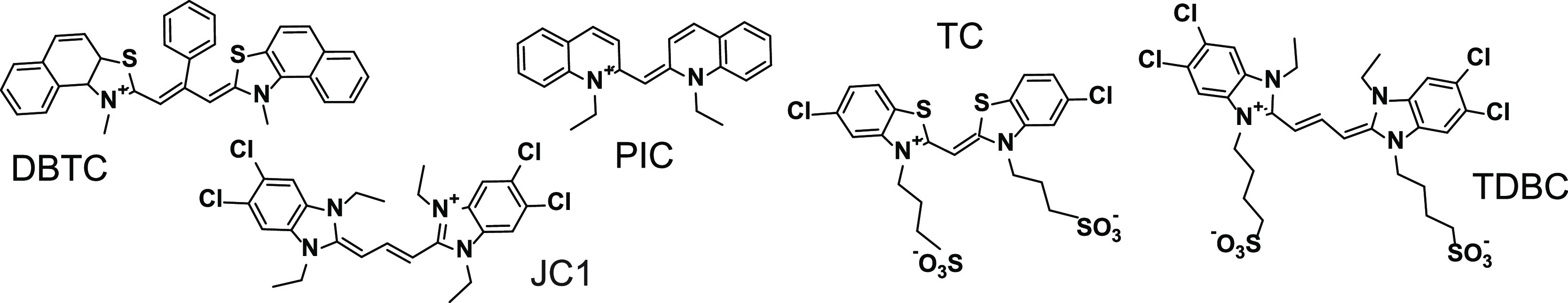
Molecular structure of the QEs most used in the preparation of CPMs. The full names are reported in Table S3.

Table 3 summarizes the different dyes used as QEs. In this case, the choices are much more heterogeneous than for the NPs materials. More than 30 molecules were used in different CPMs (see Section S2). Interestingly, five molecules emerge in this pool: DBTC, TC, TDBC, PIC, and JC1 (Figure 4), which together account for more than 60% of the CPMs reported. These molecules belong to the family of cyanines. Other dyes families used in the literature are porphyrins, carbocyanines, rhodamines, oxazines, squaraines, and triarylmethanes. In some cases, also proteins were coupled with NPs.

**Table 3:** Classification of the CPMs Based on the Chemical Nature of the QEs^a^

QEs			
molecules	CPM-S (%)	CPM-D (%)	CPM-S + CPM-D (%)
DBTC	6	0	5
JC1	13	0	10
PIC	16	12	15
TC	8	12	9
TDBC	23	35	26
others	34	42	36

family	CPM-S (%)	CPM-D (%)	CPM-S + CPM-D (%)
cyanine	79	58	74
others	21	42	26

aggregation	CPM-S (%)	CPM-D (%)	CPM-S + CPM-D (%)
monomers	23	38	27
*J*-aggregates	77	62	73

family	CPM-S (%)	CPM-D (%)	CPM-S + CPM-D (%)
cyanine	79	58	74
others	21	42	26

aggregation	CPM-S (%)	CPM-D (%)	CPM-S + CPM-D (%)
monomers	23	38	27
*J*-aggregates	77	62	73

a The percentages of samples prepared with the five molecules prevalently used are shown, together with the percentages obtained for different families of molecules (cyanines vs others) and aggregation states (monomers vs *J*-aggregates). The first two columns distinguish CPM-S and CPM-D, while the cumulative data are shown in the last column.

In most cases, these molecules are used in CPMs in the *J*-aggregate form. Indeed, excitonic transitions of dye aggregates are characterized by high dipole moment μ^60^ and low γ (20–40 meV on average compared to 100–200 meV of the monomeric QEs in our dataset). These properties favor the establishment of high *g, ℏ*Ω_R_ and CR values, according to eqs 6–8. For this reason, the exploitation of dyes capable of aggregation appears as a viable strategy for the formation of CPMs in the SC regime. Table 3 confirms this intuition revealing that in 73% of the cases, dyes forming the CPMs are aggregated.

It is worth noting that all the dye aggregates employed so far are of the *J*-type, with a single exception. Only Ni et al.^61^ reported a CPM-D plexcitonic material based on H-aggregates of HITC dyes and gold nanorods. However, the actual involvement of H-aggregates is questionable. Indeed, the absorption band of the H-aggregate overlaps with the main vibronic band of the monomer,^62^ which could be the effective species coupled with the cavity, as reported for other molecules.^63−65^

### Capping Layer: Families and Kinds of Interactions

NPs are thermodynamically unstable due to the high surface/volume ratio. The main strategy to maintain them in their dispersed state is to coat them with molecules providing a physical or electrostatic barrier against the coalescence of the core materials. It is hence not unexpected that all the plasmonic NPs used for CPM assembly feature a CL. The strength of the interaction between the NP surface and the CL ranges from weak adsorption to quasi-covalent bonds, depending on the chemical nature of the NP core and the capping molecules. In the case of noble metal NPs, a strong interaction is obtained with thiolates, while amines, phosphines, carboxylates, and halides adsorb with decreasing affinity.^66^ Added QEs can either replace the capping molecules or bind to them, depending on their nature and the nature of the capping molecules. Hence, one can easily expect that the presence of this “intermediate” layer on the surface of the NPs must influence the CPM features. On the one hand, the CL determines the assembly of the QEs on the NPs, influencing their organization, orientation, and affinity.^41^ On the other hand, it may hinder the *V*_eff_ and critically affect the number of coupled QEs.^67^

**Figure 5:**
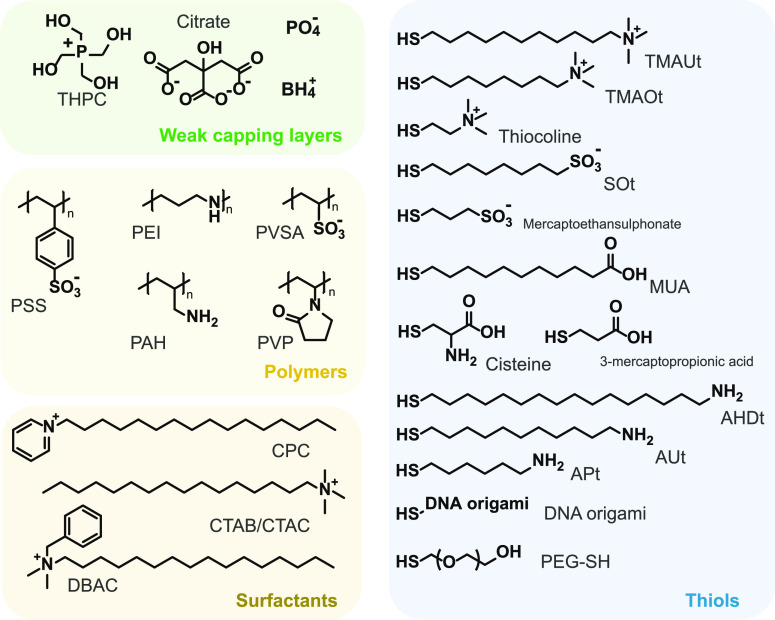
Molecular structures of the different CL molecules used in the assembly of the CPMs, grouped into four classes: weak capping layers, polymers, surfactants, and thiols. The full names of the CL molecules are reported in Table S4.

As for dye molecules, a wide variability of CL molecules is found. To find some significant correlations, we group them into four classes according to the expected interaction with the NP: weak CLs, thiols, polymers, and cationic surfactants (Figure 5). The weak CL category includes molecules that can be easily removed, or better exchanged, from the surface of NPs because their binding strength is low.^68^ Thiols include molecules featuring an −SH end-group, which is well-known to bind strongly on the Au and Ag surfaces. Polymers can feature both weak and strong interacting groups, but their adhesion to the particles is enhanced by their multivalency.^66^ Note that PEG and DNA ending with thiol groups are considered thiols and not polymers. Finally, cationic surfactants include molecules with a long aliphatic chain and a cationic head. They are also weakly bound to the surface of NPs, but they usually form a stable bilayer due to their amphiphilicity.^69^Table 4 shows that CPM-S have a uniform distribution of CL. CPM-D, instead, are prepared mostly with thiols and weak CLs (about 60%), and only 10% are capped with polymers. Indeed, deposited systems do not need stabilization against coalescence, and consequently, their preparation with NPs with weak CLs is more accessible than in the case of CPM-S.

**Table 4:** Classification of the CPMs Based on the Chemical Nature of the CL^a^

capping layers			
family	CPM-S (%)	CPM-D (%)	CPM-S + CPM-D (%)
surfactant	29	24	28
polymer	31	12	26
thiol	19	36	23
weak	21	28	23

interaction	CPM-S (%)		
direct ads. (i)	21		
with a spacer (ii)	0		
non-covalent (iii)	60		
segregation (iv)	9		
others (iii) + (iv)	9		

a The percentage of samples prepared with different families of CLs and exploiting different interactions are reported. The first two columns distinguish CPM-S and CPM-D, while the cumulative data are shown in the last column when relevant. Note that CPM-D are not classified in terms of “interaction” because in this case, the spatial proximity of QE and NP is guaranteed by their deposition and not because of mutual interactions.

**Figure 6:**
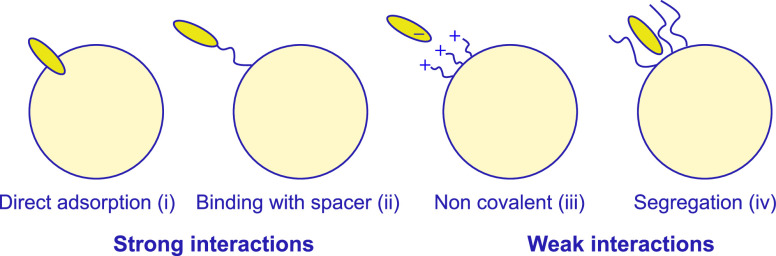
Schematization of the main interactions between NPs and dyes observed in CPMs: the direct adsorption and the covalent binding with a spacer are classified as strong interactions, while non-covalent and segregation interactions as weak interactions.

We also analyze the supramolecular interactions at play between QEs and NPs driven by the CL. We studied these interactions for CPM-S, while we considered that in CPM-D, the spatial proximity—and thus the coupling—of QE and NP is guaranteed by their deposition and not because of mutual interactions. Analyzing the literature, we schematized four different kinds of possible interactions (Figure 6): (i) the dye directly adsorbs on the metal surface; in this case, the molecules of the CL are exchanged by the dye; (ii) the dye is bound to the NP surface through a thiolate spacer (or other groups with high affinity); (iii) the dye is adsorbed on the surface of the CL through non-covalent interactions; and (iv) the dye is segregated within the CL, again as a consequence of non-covalent interactions and solvophobic effects. These different interaction scenarios influence both the number of coupled QEs and the QE affinity for the NPs. Interaction (i) places the dye at the shortest distance from the metal surface (contact), while in interactions (ii)–(iv), the distance is controlled by the CL or dye features and, in particular, by their molecular structure. The interactions (i) and (ii) can be classified as strong interactions, while the (iii) and (iv) interactions can be classified as weak. It must be noted, however, that the identification of the exact interaction occurring is not always straightforward, and in particular, the discrimination between (i) and (ii) or between (iii) and (iv) may strongly depend on peculiar features of the CL and the dye.

Considering these difficulties, we classify the interactions based on a few assumptions that may not always coincide with the ones originally proposed by the authors. In particular, it is challenging to experimentally establish whether the QEs replace the CL molecules or are adsorbed on/within them. However, for CPMs formed by nanostructures with tips and capped with citrate, the coupling with QEs was shown in many instances to smooth the edges, modifying their TEM images and extinction spectra.^41,70−74^ This evidence confirms the establishment of the direct adsorption of QEs onto the metal surface in these cases and likely also in the cases where nanostructures without edges were coated by citrate or other weak coatings. For this reason, we considered interaction (i) to be the one occurring in all the cases, where NPs are coated by a weak CL, i.e., citrate, BH_4_^+^, THPC, and PO_4_^–^. Contrariwise, in the original papers, the interactions at play are sometimes considered of the type (iii).^75,76^

Another assumption affects the definition of interactions (iii) and (iv), whose boundaries are quite labile. The *ratio* used to classify the interaction of type (iii) when the CL and the QEs had complementary interaction sites (usually opposite net charges). In contrast, interaction (iv) is assumed in the other cases, like for the CL/QEs pairs Styryl 9/M-SOt,^40^ JC1/cationic surfactant,^77−80^ and TDBC/PVP and TDBC/citrate.^81^ In some situations, interactions (iii) and (iv) are considered acting together.^19,65,73,82−84^ Interaction (iii) is assumed also in the case of dyes covalently linked to polymeric CLs.^85^ Note that interaction (ii) has not yet been reported in the literature for CPM-S, although feasible in principle. Instead, some examples of this kind of interaction were reported for CPM-D, where the dye was covalently bound to the NP surface through a spacer.^20,67,86^

Overall, Table 4 reveals that CPM-S have been assembled mainly by type (iii) interactions and that weak interactions prevail in systems dispersed in solutions (about 80% of the systems reported so far).

### Multiple Linear Regression Analysis

Following the qualitative classification of the CPMs based on the properties of their main components (NPs, QEs, and CL), we then analyze the dataset in terms of regression models as introduced in the “Materials and Methods” section. We first establish the dependence of *ℏ*Ω_R_ and CR on a single continuous variable, therefore keeping only the first two terms of eq 9. Among the available numerical variables, *ℏk* and *ℏ*γ are used to calculate CR; thus, they cannot be used as independent variables. Moreover, most CPMs have similar ω_C_ because the dataset mostly includes QEs absorbing in the spectral region between 570 and 590 nm (PIC, TDBC, and JC1). Therefore, only the size of the NPs is a relevant continuous variable. The dependence of *ℏ*Ω_R_ on quantities related to the size of the system is clearly predicted by the theoretical model of molecule-field interactions. Indeed, *ℏ*Ω_R_ is a collective quantity depending on the square root of the number of coupled emitters and the coupling strength *g*_0_ (eq 7), which scales as 1/ in an ideal cavity. The effective volume *V*_eff_ can be easily estimated when NPs are smaller than the wavelength of the light since in this case the field is confined mostly into the metal and *V*_eff_ can be approximated to the geometrical volume *V* of the NP.^89,90^ Previous analysis pointed out the dependence of *ℏ*Ω_R_ on the inverse of the effective volume^87,91^ and on the square of the long size in the case of silver nanoprisms.^92^

Here, we select as a relevant size variable the ratio , as reported in Stete et al.,^88^ where *S* is the surface area of the NP and *V* is its volume. By assuming that the number of emitters *N* is proportional to the surface area of the NP and that the effective volume can be identified with the geometrical volume of the NP, the ratio  could serve as an estimator of the factor  appearing in eq 7.

**Figure 7:**
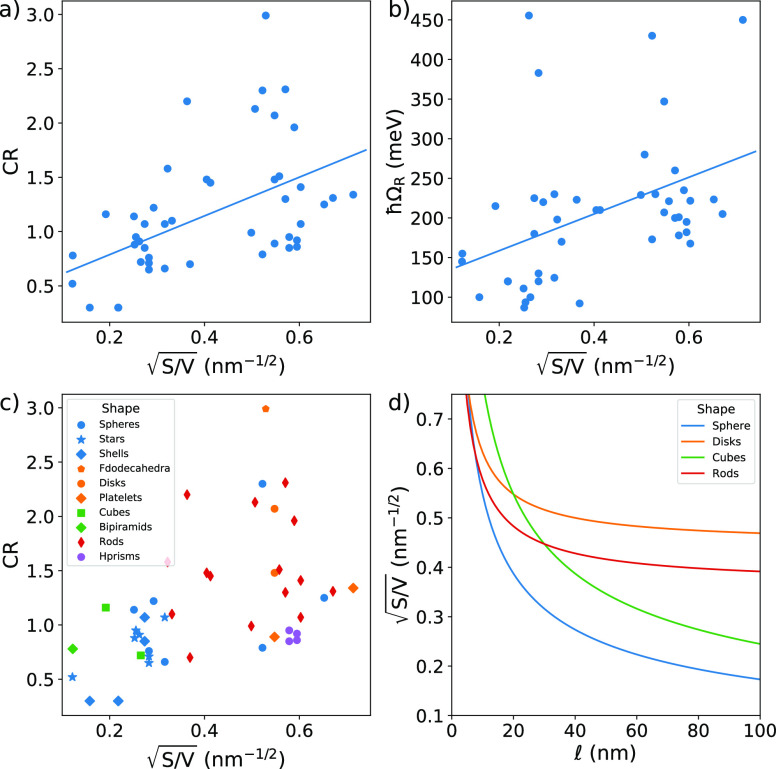
Linear regression of response variables (a) CR and (b) *ℏ*Ω_R_ as a function of  (dataset: CPM-S). The *R*^2^ and the *p*-value are, respectively, 0.26 and <0.001 for panel (a) and 0.18 and 0.003 for panel (b). (c) Multivariate plot of CR as a function of  and NP shapes (dataset: CPM-S). Shapes are distinguished by different markers, while the same color groups together similar shapes. In particular, the blue color is used for sphere-like shapes, orange for disk-like, green for cube-like, red for rods, and purple for hollow prisms. The same color code is used in (d), where a trend of  as a function of a length variable  for different shapes is plotted. The length variable  is the radius for spheres, the side for cubes, the radius of a cylinder for disks (with height *h* = 10 nm), and the height of a cylinder for rods (with radius *r* = 15 nm). More details are given in Section S5.

A linear regression on the whole dataset CPM-S + CPM-D (Table 5) shows that both *ℏ*Ω_R_ and the CR are significantly correlated with the size variable  (*p*-value < 0.05), although the model explains a limited portion of data variability (low value of *R*^2^). The correlation becomes stronger when only CPM-S are considered, while the linear trend in the CPM-D dataset is not statistically significant. For this reason, from now on, we will refer our analysis to the dataset of CPM-S if not explicitly noted otherwise. Figure 7a shows the linear regression for CR as a function of  for the CPM-S dataset. The surface-to-volume ratio decreases with increasing volume, and therefore, high CR values are favored by small NPs. A similar trend is also found for *ℏ*Ω_R_ (Figure 7b).

**Table 5:** Linear Regression Coefficients for the Response Variables CR and *ℏ*Ω_R_ Using as a Predictor the Variable  (eq 9) for the Datasets CPM-S + CPM-D, CPM-S, and CPM-D

dataset	*y*	*a*_0_ ± ε	*b*_1_ ± ε	*R*^2^	*p*-value
CPM-S + CPM-D	CR	0.48 ± 0.23	2.00 ± 0.55	0.16	<0.001
	*ℏ*Ω_R_	135 ± 34	192 ± 79	0.079	0.018
CPM-S	CR	0.43 ± 0.20	1.78 ± 0.45	0.26	<0.001
	*ℏ*Ω_R_	112 ± 32	232 ± 74	0.18	0.003
CPM-D	CR	0.23 ± 0.65	3.3 ± 1.6	0.16	0.050
	*ℏ*Ω_R_	202 ± 91	50 ± 230	0.003	0.812

Notice that the choice of as an independent size variable allows us to condensate the information about the shape of the NPs in a numerical variable. Indeed, different NPs’ shapes are characterized by specific surface-area-to-volume ratios. Figure 7c shows the data cloud colored by the NP shape. Because of the large number of possible shapes and because some shapes are clustered in a limited size range, the shape is not a good categorical variable. However, we can make some considerations based on the characteristic value of  for different shapes, which is plotted in Figure 7d for the most representative NPs as a function of one linear dimension. Further information about the evaluation of the shapes is reported in Section S5. Spheres, which by definition minimize the *S*/*V* ratio, are not effective in reaching the SC regime; the cube is a good shape but only when small sizes can be obtained, while disks and rods are more effective as the overall dimension increases. Figure 7c qualitatively confirms these considerations. Indeed, the portion of the dataset with CR > 1 is mainly populated by rod and disk-like particles. Hollow prisms, platelets, and spheres have a CR systematically lower than other shapes with a comparable surface-to-volume ratio. No cubes with small sizes are present in the data analyzed.

**Figure 8:**
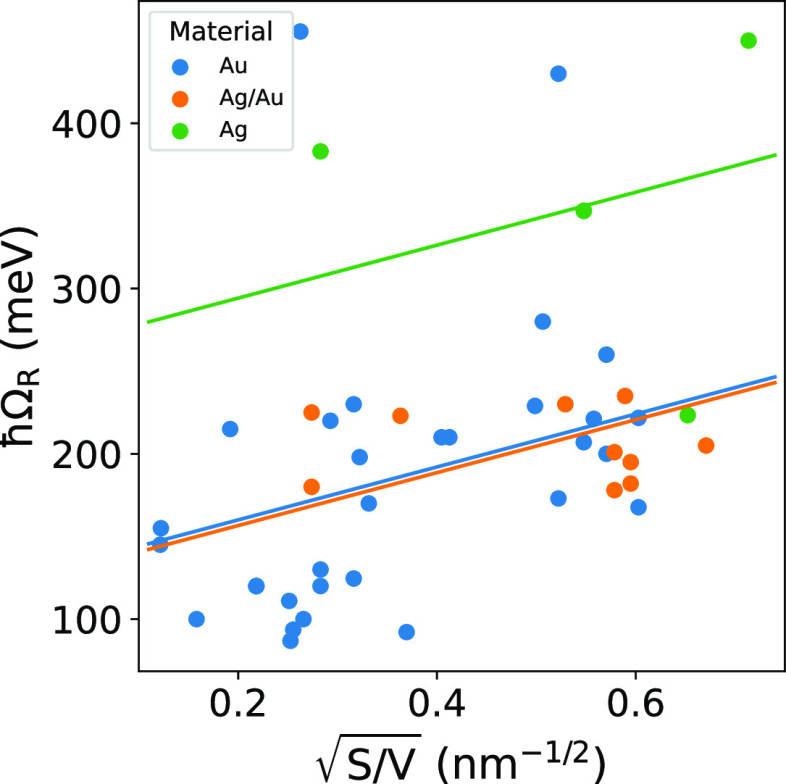
Multiple linear regression of the response variable *ℏ*Ω_R_ as a function of  and the material of the NPs (dataset: CPM-S, *R*^2^ = 0.38, *p*-value < 0.001).

**Figure 9:**
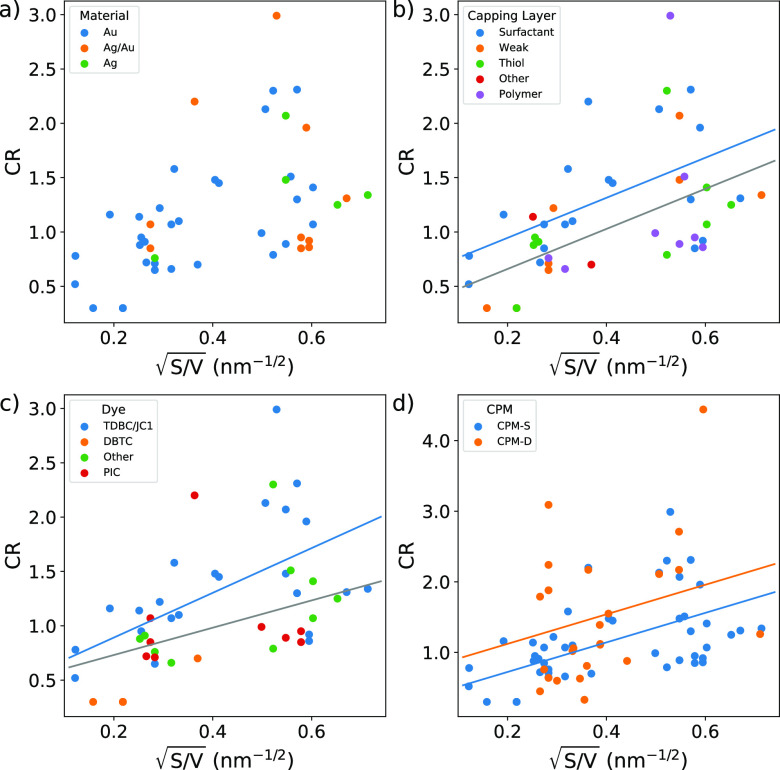
Multiple linear regressions for the response variable CR as a function of (a)  and the material of the NPs (dataset: CPM-S); (b)  and the type of CL (dataset: CPM-S, *R*^2^ = 0.32, *p*-value < 0.001); (c)  and the different dyes (dataset: CPM-S, *R*^2^ = 0.35, *p*-value < 0.001); (d)  and sample preparation (dataset: CPM-S + CPM-D, *R*^2^ = 0.23, *p*-value < 0.001).

Once established that the size of the NPs is a statistically relevant variable at least in solution samples, the key question to answer is whether other features are likely to enhance the plexcitonic character of the CPMs made of NPs of comparable size. To this end, we proceed with multivariate analysis, where the response variable is represented as a function of the size variable and colored by material (Figures 8 and 9a) and chemical nature of the CL (Figure 9b) and QE (Figure 9c) for the CPM-S dataset. The different sample preparations are compared in Figure 9d for the total dataset CPM-S + CPM-D. Correlations with the qualitative properties are quantified by the regression coefficients *c_i_* of the third term of eq 9 (while *d_ij_* = 0). Only the influence of the dye is better described by a model “with interaction” where the dye property enters the model through the fourth term of eq 9 (while *c_i_* = 0). In Section S6, we report the multivariate plots for both *ℏ*Ω_R_ and the CR for all the categorical variables and for all datasets (CPM-S, CPM-D, and CPM-S + CPM-D). Moreover, in Section S7, we also report the complete output of the statistical models highlighting significant correlations.

When multivariate plots for *ℏ*Ω_R_ are examined, the only relevant correlation emerging is with the material of the plasmonic NPs. Ag NPs have significantly higher *ℏ*Ω_R_ (around 100 meV higher, *p*-value = 0.002) than Ag/Au alloys and pure gold NPs, even if the number of reports on Ag-based plexcitons in solution is very small (Figure 8). It would be tempting to justify such dependence by invoking a higher cavity frequency (ω_C_) of silver plasmon compared to gold in morphologically similar NPs (eq 7). However, this is not the case since almost all the plexcitonic systems in the dataset have similar ω_C_ values, with no significant differences between the core materials. Indeed, the material-dependent correlation is not present in the multivariate plots for CR (Figure 9a), where instead significative correlations emerged for the dye (Figure 9b), the CL (Figure 9c), and the comparison between CPM-S and CPM-D (Figure 9d).

To investigate the correlation with the chemical nature of the dye molecules, we adopt a statistical model accounting for the interaction between the binary variable and the size variable, as represented by the last term in eq 9. The reason for this choice lies again in the simple model of field-molecule interaction, where the transition dipole moment μ of the QE is a multiplicative factor in the definition of the coupling strength (eq 7). TDBC and JC1, grouped together because of their chemical similarity (Figure 4), significantly correlate with CR (*p*-value = 0.017). For these dyes, the regression estimates an increase in the slope (*d_ij_*= 0.80 meV·nm^1/2^) compared with the other dyes in the dataset.

**Figure 10:**
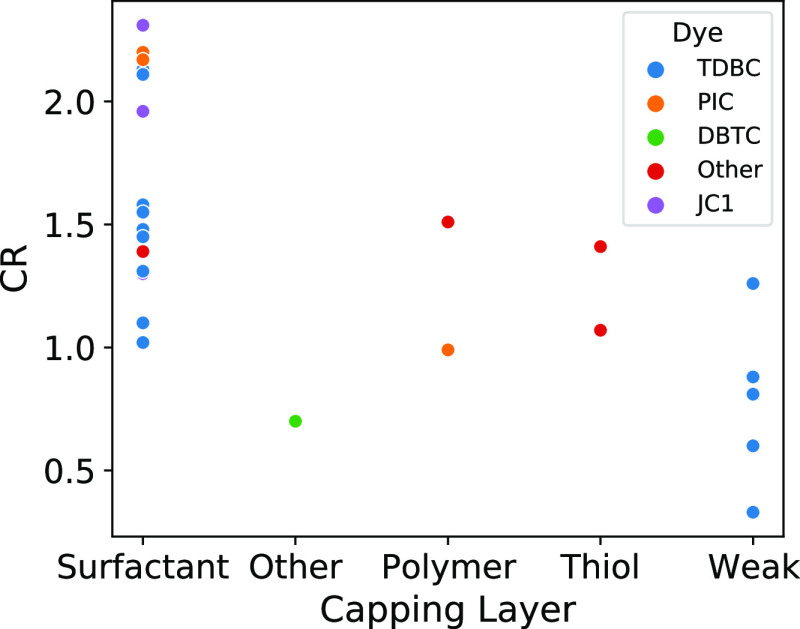
Multivariate plot of the CR as a function of the capping layer and dyes for the rod sub-ensemble (dataset: CPM-S + CPM-D).

The statistical significance of the chemical nature of the CL is more uncertain. The use of surfactants in solution samples seems to correlate with higher values of the CR, but the statistical significance is “borderline”, with *p*-value = 0.059, where the threshold is usually fixed at *p*-value < 0.05. To get more insight into the effect of the CL, we decided to investigate the sub-ensemble of rod NPs. This sub-ensemble is interesting because it contains several samples (25 data points in the total dataset CPM-S + CPM-D) with about the same value of the size variable  (between 0.4 and 0.6 nm^–1/2^) spanning from weak to SC regime. Figure 10 shows the multivariate correlations between the dye and the CL variables in this sub-ensemble. Although the size of the sub-ensemble is too limited for statistical quantification, we can appreciate how the TDBC *J*-aggregates (which in general correlates with stronger coupling in the CPM-S dataset) provide SC when associated with surfactant CL, while the ratios are lower when the same TDBC aggregates are related to weak CL. It should be noted that the weak CL here is associated with deposited samples which, in general, correlates with higher couplings. All these observations support a positive effect of surfactant CL in promoting the SC regime. There are not enough data to evaluate the combined effects of other combinations of dyes and CL systems; however, Figure S7 demonstrates that there is no strong collinearity between the two properties meaning that we can safely support the hypothesis that both TDBC/JC1 dyes and surfactant CL have a positive effect on the coupling strength independently.

The statistical analysis based on the nature of the supramolecular interactions, as illustrated in Figure 6, was not performed because most of the samples fall in the “non-covalent” (iii) interactions. However, significant differences were found when comparing CPM-S and CPM-D datasets. By including the deposited samples and looking for a correlation between CR and sample preparation (Figure 9d), the deposition correlates significantly (*p*-value = 0.021) with a higher CR, with an estimate of +0.40 meV compared to solution samples of comparable size. However, this result depends sensibly on the data reported by Rodarte et al.,^67^ featuring high CR in correspondence of a value of  around 0.3 nm^–1/2^. Because the common origin of the data is a biasing factor in the analysis, we believe that the correlation between deposition and SC should be further supported by additional experiments.

### Final Remarks

In an attempt to rationalize the design principles to effectively prepare strongly coupled colloidal plexcitonic nanohybrids, our approach consisted of (i) the preparation of a dataset by collecting all the CPMs reported so far in the literature (to the best of our knowledge); (ii) a first qualitative analysis of the dataset to determine the most recurring combinations of NPs, QEs, and CLs; and finally (iii) a statistical analysis to quantify correlations and suggest possible guidelines for the preparation of CPMs with tailored properties.

The first analysis of the dataset, despite its simple qualitative character, provided relevant information on the nature of the components more suited to produce plexcitonic hybrids in the SC regime since most of the NP/QE/CL combinations that were ineffective in forming plexcitons were likely not published and did not enter in the dataset. On the other hand, one should also consider that the recurrent presence of selected components in the dataset could be due not only to the effectiveness in the achievement of the SC regime but also to other more contingent factors, such as cost, availability, and synthetic accessibility of the materials.

That said, the first relevant information provided by the dataset classification is that the most commonly used NPs are gold spheres and rods (Table 2), while other geometries (bipyramids, cubes, platelets, shells, stars, etc.) and materials (Ag or Ag/Au alloy) are less explored. Most likely, this is because spheres and rods are the most widely studied plasmonic NPs, and their synthesis is simple and well-attested in the literature. However, statistical analysis revealed that potentially also other shapes (in particular disk-like shapes) can in principle better optimize the geometrical parameters to achieve SC.

Another piece of information extracted from Table 3 is that the formation of plexcitonic systems with monomeric molecules is relatively infrequent. There are two main reasons which could explain this evidence. First, it is now well-established that monomeric molecules are characterized by lower values of μ and higher values of γ with respect to their aggregates.^93,94^ According to the model of field-molecule interaction described before (eq 7), this intrinsically leads to smaller *ℏ*Ω_R_ and CR values. Second, monomeric molecules have lower affinities for the NPs than the *J*-aggregates, which have a greater number of possible anchoring points.^42^ As a consequence, the identification of plexcitonic bands in the extinction spectra of CPM-S prepared with monomeric dyes is considerably more challenging since they might be (partially) hidden by the strong signal due to the unbound monomers free in solution.^82,95^ Consistently, in the case of CPM-D, which do not rely on affinity since the QE/NP interaction is driven by deposition, the percentage of plexcitonic systems based on monomeric dyes is larger, increasing from 20% in CPM-S to 40% in CPM-D.

We also examined the possible effect of the choice of specific CLs. In particular, we identified four classes of CLs (weak, thiols, cationic surfactants, and polymers) and four kinds of interactions that can promote the coupling between the NPs and the dyes (direct adsorption, binding with a spacer, non-covalent interactions, and segregation). While the distribution of CLs seems to be equal in the literature, most CPM-S are prepared using CLs able to promote non-covalent interactions and segregation, both classified as weak supramolecular interactions.

This finding further supports the crucial importance of supramolecular equilibrium parameters such as affinity in establishing specific coupling regimes. Multiple linear regression models were used to quantify the correlations between the design properties of CPMs and the effective establishment of the SC regime. Being intrinsically “data driven” and not grounded on a specific quantum Hamiltonian, the statistical models allowed us to extract significant correlations despite the inhomogeneities of real systems (e.g., uneven coverage of the NP, variability in NP shape and size, non-uniform coupling strength between plasmonic field and molecular emitters, etc.).

The most robust correlation that the analysis reveals is between CR and the size parameter  of the NPs, in agreement with the predictions of the model of field–molecule interaction, showing that *ℏ*Ω_R_ (and, thus, CR) can be maximized by increasing the number *N* of QEs coupled to the NP.^41,71,96^ However, it should be noted that, depending on the NP’s geometry, it is not possible to achieve the SC regime in all cases since once the *V*_eff_ is spatially saturated by molecules, *ℏ*Ω_R_ cannot increase anymore. The fitting model estimated that the likelihood of SC (CR > 1) in CPMs is enhanced when the morphology of the NPs is such that  > 0.32 (Figure 7). Geometrical considerations (Figure 7) reveal that some shapes can fulfill this condition more easily, in particular rods and disk-like shapes. The literature inspection confirms that all the CPMs prepared using NPs with these shapes, excluding hollow prisms and platelets, are characterized by CR values above 1, corroborating our conclusions about the influence of NP shapes in the SC. Sphere-like shapes (spheres, shells, and stars), even if they are the most represented in the dataset, generally have lower  values. In order to maximize the surface-area-to-volume ratio, spheres and cubes would require small dimensions, which are not always ideal for preparing CPMs. The dimensions of the NPs do not impact only the  parameter but also play a fundamental role in determining the plasmon resonance wavelength: generally, the smaller the dimensions, the more blue-shifted the plasmon resonance. This might limit the range of dyes with transition frequencies in resonance with the NPs’ plasmon. Overall, rods and disk-like particles seem to be the most versatile and convenient shapes since they allow to achieve the  > 0.32 condition in a vast range of dimensions, potentially reaching the SC regime for all QEs that absorb in the visible spectrum.

Among the QEs analyzed, TDBC and JC1 aggregates are the most performative in achieving SC. This was already justified by invoking the smaller γ and higher values of μ of aggregated species with respect to monomers. Comparing the outcome of different *J*-aggregates is instead less trivial. Two independent studies reported that CPM-S prepared with TDBC *J*-aggregates were more coupled than those prepared with PIC *J*-aggregates. This evidence was tentatively explained considering the different tendency to aggregate of the dyes in solution,^41^ and the overall number of molecules that the NPs can host on their surface.^83^ In addition, one should also consider that the *J*-aggregates of different molecules can have different transitions dipole moments. However, estimating this parameter is challenging, considering that this value might differ significantly from *J*-aggregates in solution and on the surface of the NPs.

Among the CL families, the statistical analysis indicates the cationic surfactants as the most favorable for the SC regime. This somewhat contrasts with the chemical intuition, which suggests that the most suitable CL to promote SC between the NP and the dye should be a weak CL because it can be easily replaced by the QE molecules on the NP surface. One can speculate that the surfactant CL does not have only a passive role in NP stabilization but also an active function in recruiting the QEs. The cationic surfactants, indeed, produce a mobile bilayer onto the NP’s surface because of their amphiphilic nature. This layer could accommodate the apolar residues of the QEs, increasing the overall amount of QEs within the *V*_eff_ compared to weak CLs. The comparison reported in Figure 10 supports this interpretation. A more comprehensive evaluation of the role of the CL should also take into account additional variables such as the thickness of the CL and its capability to load dyes molecules in the *V*_eff_. However, such information was generally not available in the literature analyzed, which limits the scope of analysis based on these parameters.^62^ As a last observation, CPM-D generally seems to have higher CR values than CPM-S, which can be justified considering that deposited systems do not depend on supramolecular parameters like affinity, which are crucial in solution. This, in turn, might suggest that deposited nanosystems might become a winning strategy to simplify the preparation of SC plexciton systems.

## Conclusions

CPMs are nanosystems with promising properties in the context of polaritonic materials. Nonetheless, about 40% of the CPMs reported so far in the literature do not fulfill the SC condition, limiting their effective exploitation and suggesting the need for a deeper understanding of the design principles regulating their properties. To contribute to filling this gap, we prepared and analyzed a dataset of all the CPMs reported so far in the literature to our knowledge. We were able to identify some meaningful structure-to-properties relationships, which could prompt the development of a new generation of CPMs and move forward the preparation of plexcitons from the current trial-and-error approach to a rational design one.

The dataset was first analyzed qualitatively to identify which components (NPs, QEs, and CLs) were more frequently employed to prepare CPMs. Then, a multiple linear regression analysis was applied to correlate various design principles to achieve an SC regime and the effective formation of hybrid plexcitonic states.

It was found that the most promising CPMs have at least one of the following features:

(i) The constituent NPs must have high  values. As a rule of thumb, CPMs are in the SC regime when they are formed from NPs having  > 0.32. These values are easily obtained with rods, disks, and flat dodecahedra.
(ii) At least for CPMs in solution, the constituent dyes must be able to form *J*-aggregates on the NPs’ surface (among the systems reported, TDBC and JC1 are the most performative) because this leads to a favorable combination of μ, γ, and affinity.
(iii) The CL for CPM-S must preferentially be composed of cationic surfactants. The role of the CL is by far the most elusive to be assessed with the available data. In this case, we could only speculate that this CL family not only is sufficiently mobile to be easily displaced by QEs in the *V*_eff_ of the NP, but can also actively recruit the QEs within the *V*_eff_.
(iv) Many requirements found essential for CPM-S are relaxed when the nanosystems get deposited in CPM-D because supramolecular interactions at play in solution become less relevant.

Although some of these guidelines could be directly inferred from the theoretical models and have already been, more or less consciously, applied in the preparation of strongly coupled nanosystems (as, for example, the beneficial use of *J*-aggregates), the identification of these clear guidelines is the first step toward a more careful and informed approach to the preparation of plexcitonic nanohybrids and a better understanding of the complex and still unexplored supramolecular interactions driving the formation of such systems in solution.

In addition, promising future directions have also been identified. For example, it emerged clearly that a crucial step in the design of CPMs is the control of the supramolecular interactions at play between NPs, CLs, and QEs, despite this aspect having been scarcely considered.^41,42,82,97^ The molecular details of the interactions between QE and CL are unclear. Thus, it is challenging to predict which interactions are the most suitable to increase affinity, and, in turn, *ℏ*Ω_R._

In addition, the preparation of deposited samples (CPM-D) is particularly versatile. On the one hand, the deposition allows avoiding several limitations found for supramolecular assemblies in solution and thus exploring a vaster range of combinations of NPs, QEs (even in monomeric form), and CLs. On the other hand, it solves some of the major drawbacks of NPs dispersed in solution, i.e., their instability due to etching and their natural tendency of precipitation for gravity.

Another interesting alternative route for preparing CPM-S is to covalently bind the QEs to the surface of the NPs using a spacer [interaction (ii), Figure 6]. This strategy would in principle allow for the coupling of different kinds of QEs, also in monomeric form. Although in principle feasible, as proposed for CPM-D,^20,67,86^ this approach has not yet been attempted in the literature for CPM-S.

In conclusion, with this paper, we rationalized the CPMs’ design and identified some properties that might enhance their performances and versatility. By identifying clear structure-to-properties relationships, we can finely tailor the light–matter coupling temporally and spatially, even at the nanometric scale. This will open numerous scenarios for applications in many fields, including polaritonic chemistry, nanophotonics, quantum technologies, and energy conversion technology.

## Supporting Information Available

Colloidal plexcitonic material dataset, molecular nomenclature and abbreviations, and definition of the dataset and multiple linear regression (PDF)
