# Supplementary material for: Identification of Design Principles for the Preparation of Colloidal Plexcitonic Materials
Source: Langmuir. 2023 Aug 29;39(36):12793–806. doi: 10.1021/acs.langmuir.3c01642 (PMC10501205; doi:10.1021/acs.langmuir.3c01642)
Supplement: Supplementary file 1 — la3c01642_si_001.pdf [file la3c01642_si_001.pdf]

ELECTRONIC SUPPORTING INFORMATION

Identification of design principles for the preparation of colloidal plexcitonic materials

Nicola Peruffo,[1†] Matteo Bruschi,[1] Barbara Fresch,[1,2] Fabrizio Mancin,[1,] Elisabetta Collini[1,2,*]*

[1] Department of Chemical Sciences, University of Padova, via Marzolo 1, 35131 Padova, Italy

[2] Padua Quantum Technologies Research Center, via Gradenigo 6/A, 35122 Padova, Italy

[†] Present address: Department of Chemistry and Molecular Biology, University of Gothenburg, Medicinaregatan 19, 41390 Gothenburg, Sweden

* elisabetta.collini@unipd.it, fabrizio.mancin@unipd.it

Index

S1. Colloidal Plexcitonic Materials (CMPs) dataset 2

S2. Molecular nomenclature and abbreviations 10

S3. Additional notes about the definition of the dataset 11

S4. Multiple linear regression 12

S5. Surface area and volume for different NP shapes 13

S6. Bivariate Plots 14

S7. Linear Regression Models 20

S8. References 25

S1. Colloidal Plexcitonic Materials (CMPs) dataset

Table S1. Dataset of CPM-S. In the headers, Mat. stands for material, Fam. for family, J for J-aggregate, M for monomeric form, Int. for interaction. The full names of dye molecules are reported in Table S3. The dye families are indicated with abbreviations: Cy=Cyanines, Cc=Carbocyanines, O=Oxazines, P=proteins, Po= Porphyrins, Rh=Rhodamines, Sq=Squaraines and T=triarylmethanes. The full names of the CL molecules are reported in Table S4. The interactions are classified as in the main text: (i) = direct dye-metal interaction; (iii)= electrostatic interaction; (iv)= segregation. When in the original reference the CL was not indicated, the CL and Interactions entries are classified as “Other”. The coupling parameters $\hbar\Omega_R$, $\hbar\gamma$, and $\hbar k$ are reported in meV. S (=NP’s surface area) is reported in 10^3 x nm^2, V (=NP’s volume) is reported in 10^3 x nm^3.

**Chronological**				**NPs**		**Dye**			**CL**		**Int.**	**Coupling parameters**				**Size parameters**		
System #	Paper #	1st author & year	Ref	Mat.	Shape	Name	Fam.	J/M	Mat.	Fam.	Int.	$\hbar\Omega_R$	ħk/2	ħγ/2	CR	S	V	sqrt (S/V)
1	1	Kometani 2001	1	Au	Sphere	TC	Cy	J	BH^{4+}	Weak	i	High δ	-	-	-	-	-	-
2	1	Kometani 2001	1	Ag	Sphere	TC	Cy	J	BH^{4+}	Weak	i	High δ	-	-	-	-	-	-
3	1	Kometani 2001	1	Ag-Au	Sphere	TC	Cy	J	BH^{4+}	Weak	i	High δ	-	-	-	-	-	-
4	2	Sato 2001	2	Ag	Sphere	TC(Et)	Cy	J	BH^{4+}	Weak	i	High δ	-	-	-	-	-	-
2	3	Hranisavljevic 2002	3	Ag	Sphere	TC	Cy	J	BH^{4+}	Weak	i	High δ	-	-	-	-	-	-
1	4	Wiederrecht 2004	4	Au	Sphere	TC	Cy	J	BH^{4+}	Weak	i	High δ	-	-	-	-	-	-
2	4	Wiederrecht 2004	4	Ag	Sphere	TC	Cy	J	BH^{4+}	Weak	i	High δ	-	-	-	-	-	-
2	5	Yoshida 2008	5	Ag	Sphere	TC	Cy	J	BH^{4+}	Weak	i	High δ	-	-	-	-	-	-
5	6	Fofang 2008	6	Au	Shell	DBTC	Cy	J	THPC	Weak	i	120	368	26	0.30	49.9	1047.4	0.22
5	6	Fofang 2008	6	Au	Shell	DBTC	Cy	J	THPC	Weak	i	100	305	26	0.30	181.0	7238.2	0.16
6	6	Fofang 2008	6	Au	Shell	DBTC	Cy	J	Mercapto etan sulfonate	Thiol	iii	120	368	26	0.30	49.9	1047.4	0.22
7	7	Ni 2008	7	Au	Rods	HITC	Cc	M	PSS	Polymer	iii	Low Ka	-	-	-	-	-	-

Chronological				NPs		Dye			CL		Int.	Coupling parameters				Size parameters		
System #	Paper #	1st author & year	Ref	Mat.	Shape	Name	Fam.	J/M	Mat.	Fam.	Int.	$\hbar\Omega_R$	ħk/2	ħγ/2	CR	S	V	sqrt (S/V)
1	8	Yoshida 2009	8	Au	Sphere	TC	Cy	J	BH^{4+}	Weak	i	High δ	-	-	-	-	-	-
8	8	Yoshida 2009	8	Au	Sphere	TC	Cy	J	TMAUt	Thiol	iii	High δ	-	-	-	-	-	-
9	8	Yoshida 2009	8	Au	Sphere	SO-PIC	Cy	J	TMAUt	Thiol	iii	High δ	-	-	-	-	-	-
10	9	Yoshida 2009	9	Au	Rods	TC	Cy	J	TMAUt	Thiol	iii	High δ	-	-	-	-	-	-
11	9	Yoshida 2009	9	Au	Rods	TC(Ph)	Cy	J	TMAUt	Thiol	iii	222	137	20	1.41	1.7	4.8	0.60
12	9	Yoshida 2009	9	Au	Rods	TC(Et)	Cy	J	TMAUt	Thiol	iii	168	137	20	1.07	1.7	4.8	0.60
13	10	Juluri 2009	10	Au	Rods	DBTC	Cy	J	Not reported			92	106	26	0.70	11.8	86.6	0.37
14	11	Choi 2009	11	Au	Sphere	Cc	P	M	3-mercapto propionic acid	Thiol	iii	Double $\hbar\Omega_R$	-	-	-	-	-	-
15	12	Lekeufauck 2010	12	Au	Sphere	TDBC	Cy	J	Citrate	Weak	i	220	165	15	1.22	15.4	179.6	0.29
16	13	Yoshida 2010	13	Ag	Sphere	TC	Cy	J	TMAUt	Thiol	iii	High δ	-	-	-	-	-	-
17	13	Yoshida 2010	13	Ag	Sphere	OC	Cy	J	TMAUt	Thiol	iii	223	179	?	1.25	0.6	1.5	0.65
18	14	Ni 2010	14	Au	Rods	R640	Rh	M	PSS	Polymer	iii	Low Ka	-	-	-	-	-	-
19	14	Ni 2010	14	Au	Rods	CV	O	M	PSS	Polymer	iii	Low Ka	-	-	-	-	-	-
20	14	Ni 2010	14	Au	Rods	MG	T	M	PSS	Polymer	iii	Low Ka	-	-	-	-	-	-
21	14	Ni 2010	14	Au	Rods	O720	O	M	PSS	Polymer	iii	Low Ka	-	-	-	-	-	-
22	14	Ni 2010	14	Au	Rods	DOTCI	Cc	M	PSS	Polymer	iii	Low Ka	-	-	-	-	-	-
23	14	Ni 2010	14	Au	Rods	O725	O	M	PSS	Polymer	iii	Low Ka	-	-	-	-	-	-
24	14	Ni 2010	14	Au	Rods	HITC	Cc	M	PSS	Polymer	iii	Low Ka	-	-	-	-	-	-

Chronological				NPs		Dye			CL		Int.	Coupling parameters				Size parameters		
System #	Paper #	1st author & year	Ref	Mat.	Shape	Name	Fam.	J/M	Mat.	Fam.	Int.	$\hbar\Omega_R$	$\hbar k/2$	$\hbar\gamma/2$	CR	S	V	sqrt (S/V)
25	14	Ni 2010	14	Au	Rods	MB	T	M	PSS	Polymer	iii	Low Ka	-	-	-	-	-	-
26	15	Hao 2011	15	Au	Rods	TC(Et)	Cy	J	CTAB	Surfactant	iii	190	92	20	1.70	No TEM		
5	16	Fofang 2011	16	Au	Shell	DBTC	Cy	J	THPC	Weak	iii	120	368	26	0.30	49.9	1047.4	0.22
27	17	Balci 2013	17	Ag	Prism	TDBC	Cy	J	Citrate	Weak	i	447	275	15	1.54	No TEM		
28	18	Melnikau 2013	18	Au	Stars	JC1	Cy	J	CTAB+PEI	Polymer	iii/iv	260	267	12	0.93	No TEM		
29	18	Melnikau 2013	18	Au	Stars	JC1+ S2165	Cy	J/M	CTAB+PEI	Polymer	iii/iv	Double $\hbar\Omega_R$ (180+110)	-	-	-	-	-	-
27	19	Balci 2014	19	Ag	Prism	TDBC	Cy	J	Citrate	Weak	i	447	275	15	1.54	No TEM		
30	20	DeLacy 2015	20	Au	Platelets	PIC	Cy	J	Citrate+ PVP	Polymer	iii	207	221	13	0.89	3.8	12.6	0.55
31	21	Nan 2015	21	Au	Rods	IR806	Cc	M	PAH	Polymer	iii	221	100	46	1.51	2.5	8.0	0.56
32	22	Fales 2015	22	Au	Sphere	PIC	Cy	J	PVSA	Polymer	iii	High δ	-	-	-	-	-	-
33	22	Fales 2015	22	Au	Rods	PIC	Cy	J	PVSA	Polymer	iii	129	110	13	1.06	No TEM		
27	23	Balci 2016	23	Ag	Prism	TDBC	Cy	J	Citrate	Weak	i	447	275	15	1.54	No TEM		
34	24	Melnikau 2016	24	Au	Rods	JC1	Cy	J	CTAB	Surfactant	iv	200	142	12	1.30	2.0	6.0	0.57
34	25	Simon 2016	25	Au	Rods	JC1	Cy	J	CTAB	Surfactant	iv	260	100	12	2.31	2.0	6.0	0.57
35	26	Hazra 2016	26	Ag-Au	Rings	PIC	Cy	J	CTAB	Surfactant	iii	High δ	-	-	-	-	-	-
36	27	Liu 2017	27	Ag-Au	Cubes	PIC	Cy	J	CTAB	Surfactant	iii	223	89	13	2.20	10.7	81.2	0.36
37	28	Das 2017	28	Ag-Au	Rings	PIC	Cy	J	CTAB	Surfactant	iii	193	204	11	0.90	No TEM		
38	29	Melkinau 2017	29	Ag-Au	Rods	JC1	Cy	J	DBAC	Surfactant	iv	175	141	12	1.14	No TEM		

Chronological				NPs		Dye			CL		Int.	Coupling parameters				Size parameters		
System #	Paper #	1st author & year	Ref	Mat.	Shape	Name	Fam.	J/M	Mat.	Fam.	Int.	$\hbar\Omega_R$	$\hbar k/2$	$\hbar\gamma/2$	CR	S	V	sqrt (S/V)
39	30	Thomas 2018	30	Ag	Sphere	FITC	Rh	M	PVP	Polymer	iii	383	415	90	0.76	17.7	220.9	0.28
40	30	Thomas 2018	30	Au	Rods	PIC	Cy	J	PSS	Polymer	iii/iv	229	215	17	0.99	3.0	12.1	0.50
41	31	Walters 2018	31	Au	Sphere	TC(Et)'	Cy	J	Thiocoline +PAH+PVP +SI	Polymer	iii/iv	125	175	15	0.66	11.3	113.1	0.32
42	32	Kirschner 2018	32	Au	Bipiramids	DBTC	Cy	J	CTAB+ CTAC	Surfactant	iii	120	67	26	1.29	No TEM		
43	33	Song 2019	33	Au	Cubes	PIC	Cy	J	Cl^-	Surfactant	iii	100	127	13	0.72	43.4	614.1	0.27
44	34	Melnikau 2019	34	Ag-Au	Rods	JC1+ S2165	Cy	J	DBAC	Surfactant	iii/iv	Double $\hbar\Omega_R$ (175+163)	-	-	-	-	-	-
42	35	Kirschner 2019	35	Au	Bipiramids	DBTC	Cy	J	CTAB+ CTAC	Surfactant	iii	120	67	26	1.29	No TEM		
45	36	Balci 2019	36	Ag	Disks	TDBC	Cy	J	Citrate	Weak	i	347	219	15	1.48	3.8	12.6	0.55
46	37	Sun2019	37	Ag-Au	Shell	PIC	Cy	J	CTAB	Surfactant	iii	225	202	9	1.07	20.1	268.1	0.27
47	37	Sun2019	37	Ag-Au	Shell	PIC	Cy	J	CTAB	Surfactant	iii	180	202	9	0.85	20.1	268.1	0.27
48	38	Guvenc 2020	38	Ag	Disks	TDBC	Cy	J	Citrate	Weak	i	347	153	15	2.07	3.8	12.6	0.55
49	38	Guvenc 2020	38	Ag-Au	Rings	TDBC	Cy	J	Citrate	Weak	i	331	170	15	1.79	No TEM		
37	39	Das 2020	39	Ag-Au	Rings	PIC	Cy	J	CTAB	Surfactant	i	178	200	11	0.85	3.8	11.4	0.58
50	39	Das 2020	39	Ag-Au	Rings	TDBC	Cy	J	CTAB	Surfactant	iii	195	200	13	0.92	3.1	8.8	0.59
51	39	Das 2020	39	Ag-Au	Rings	PIC	Cy	J	PSS	Polymer	iii	201	200	11	0.95	3.8	11.4	0.58
52	39	Das 2020	39	Ag-Au	Rings	TDBC	Cy	J	PSS	Polymer	iii/iv	182	200	13	0.86	3.1	8.8	0.59
53	40	Kumar 2020	40	Au	Rods	TDBC	Cy	J	CTAB	Surfactant	iii	280	119	13	2.13	2.8	10.9	0.51

Chronological				NPs		Dye			CL		Int.	Coupling parameters				Size parameters		
System #	Paper #	1st author & year	Ref	Mat.	Shape	Name	Fam.	J/M	Mat.	Fam.	Int.	$\hbar\Omega_R$	ħk/2	ħγ/2	CR	S	V	sqrt (S/V)
53	40	Kumar 2020	40	Au	Rods	TDBC	Cy	J	CTAB	Surfactant	iii	210	129	13	1.48	6.8	41.7	0.40
53	40	Kumar 2020	40	Au	Rods	TDBC	Cy	J	CTAB	Surfactant	iii	170	142	13	1.10	15.0	136.7	0.33
54	41	Hendel 2020	41	Ag-Au	Rods	JC1	Cy	J	CTAB	Surfactant	iv	235	108	12	1.96	1.6	4.6	0.59
55	41	Hendel 2020	41	Au	Sphere	JC1	Cy	J	Not reported			111	85	12	1.14	28.4	448.9	0.25
56	42	Li 2020	42	Ag-Au	Cubes	TDBC	Cy	J	CPC	Surfactant	iii	162	124	13	1.19	No TEM		
57	42	Li 2020	42	Ag-Au	Rings	TDBC	Cy	J	CPC	Surfactant	iii	186	111	13	1.51	No TEM		
42	43	Kirshner 2020	43	Au	Bipiramids	DBTC	Cy	J	CTAB+ CTAC	Surfactant	iii/iv	120	67	26	1.29	No TEM		
58	44	Mohankumar 2020	44	Au	Rods	SQ	Sq	M	PSS	Polymer	iii/iv	Low Ka	-	-	-	-	-	-
59	44	Mohankumar 2020	44	Ag	Sphere	Cy	Cy	M	Citrate	Weak	i	Low Ka	-	-	-	-	-	-
60	44	Mohankumar 2020	44	Au	Rods	Cy5	Cy	M	PSS	Polymer	iii	Low Ka	-	-	-	-	-	-
D14	45	Stete2020	45	Au	Rods	TDBC	Cy	J	Citrate	Weak	i	227	62	15	2.95	No TEM		
61	46	Krivenkov 2021	46	Ag	Platelets	JC1	Cy	J	Citrate	Weak	i	450	325	12	1.34	1.5	2.9	0.71
62	47	Peruffo 2021	47	Au	Sphere	TPPS	Po	M	TMAOt	Thiol	iii	173	175	43	0.79	1.5	5.6	0.52
63	47	Peruffo 2021	47	Au	Sphere	TPPS	Po	J	TMAOt	Thiol	iii	430	175	12	2.30	1.5	5.6	0.52
64	48	Peruffo 2021	48	Au	Stars	Styryl 9M	Cc	M	SOt	Thiol	iv	456	188	311	0.91	25.4	369.1	0.26
65	48	Peruffo 2021	48	Au	Stars	Cy75	Cy	J	TMAOt	Thiol	iii	87	84	15	0.88	27.8	434.9	0.25
66	48	Peruffo 2021	48	Au	Stars	PIC	Cy	J	Citrate	Weak	i	130	171	13	0.71	17.7	220.9	0.28
67	48	Peruffo 2021	48	Au	Stars	TDBC	Cy	J	Citrate	Weak	i	120	171	15	0.65	17.7	220.9	0.28
68	48	Peruffo 2021	48	Au	Stars	TDBC	Cy	J	TMAOt	Thiol	iii	94	84	15	0.95	26.6	407.7	0.26

Chronological				NPs		Dye			CL		Int.	Coupling parameters				Size parameters		
System #	Paper #	1st author & year	Ref	Mat.	Shape	Name	Fam.	J/M	Mat.	Fam.	Int.	ħΩ$_R$	ħk/2	ħγ/2	CR	S	V	sqrt (S/V)
27	49	Finkelstein - Shapiro 2021	49	Ag	Prism	TDBC	Cy	J	Citrate	Weak	i	High δ	-	-	-	-	-	-
53	50	Guo 2021	50	Au	Rods	TDBC	Cy	J	CTAB	Surfactant	iii	198	110	15	1.58	17.1	164.9	0.32
69	51	Melnikau 2021	51	Au	Stars	JC1	Cy	J	CTAB	Surfactant	iv	230	202	14	1.07	11.3	113.1	0.32
70	52	Balci 2021	52	Ag-Au	Dodecahedra	TDBC	Cy	J	Citrate+PVP	Polymer	iv	230	61	16	2.99	5.5	19.6	0.53
71	53	Zhu 2021	53	Ag-Au	Rods	TDBC	Cy	J	DNA origami+ CPC	Surfactant	iii	205	140	16	1.31	2.8	6.3	0.67
56	54	Wu 2021	54	Ag-Au	Cuboids	TDBC	Cy	J	CPC	Surfactant	iii	214	149	23	1.24	No TEM		
53	55	Kumar 2022	55	Au	Rods	TDBC	Cy	J	CTAB	Surfactant	iii	210	130	15	1.45	2.8	6.2	0.67
72	55	Kumar 2022	55	Au	Cubes	TDBC	Cy	J	CTAC	Surfactant	iii	215	170	15	1.16	0.7	20.0	0.19
73	55	Kumar 2022	55	Au	Bipiramids	TDBC	Cy	J	CTAB	Surfactant	iii	155	185	15	0.78	4.5	300	0.12
74	55	Kumar 2022	55	Au	Stars	TDBC	Cy	J	CTAB	Surfactant	iii	145	264	15	0.52	4.6	310	0.12
62	56	Peruffo 2022	56	Au	Sphere	TPPS	Po	M	TMAOt	Thiol	iii	173	175	43	0.79	1.5	5.6	0.52
63	56	Peruffo 2022	56	Au	Sphere	TPPS	Po	J	TMAOt	Thiol	iii	430	175	12	2.30	1.5	5.6	0.52
62	57	Peruffo 2022	57	Au	Sphere	TPPS	Po	M	TMAOt	Thiol	iii	173	175	43	0.79	1.5	5.6	0.52
63	57	Peruffo 2022	57	Au	Sphere	TPPS	Po	J	TMAOt	Thiol	iii	430	175	12	2.30	1.5	5.6	0.52
75	58	Melnikau 2022	58	Au	Rods	JC1	Cy	J	CTAB	Surfactant	Iv	230	202	13.5	No TEM			

Table S2. Dataset of CPM-D. The CPM-D literature and systems are differentiated from CPM-S listing them as D1, D2, etc. In the headers, Mat. stands for material, Fam. for family, J for J-aggregate, M for monomeric form, Int. for interaction. The full names of dye molecules are reported in Table S3. The dye families are indicated with abbreviations: Cy=Cyanines, Cc=Carbocyanines, O=Oxazines, P=proteins, Po= Porphyrins, Rh=Rhodamines, Sq=Squaraines and T=triarylmethanes. The full names of the CL molecules are reported in Table S4. The interactions are classified as in the main text: (i) = direct dye-metal interaction; (iii)= electrostatic interaction; (iv)= segregation. When in the original reference the CL was not indicated, the CL and Interactions entries are classified as "Other". The coupling parameters $\hbar\Omega_R$, $\hbar\gamma$, and $\hbar k$ are reported in meV. S (=NP's surface area) is reported in 10^3 x nm^2, V (=NP's volume) is reported in 10^3 x nm^3.

Chronological				NPs		Dye			CL		Coupling parameters				Size parameters		
System #	Paper #	1st author & year	Ref.	Mat.	Shape	Name	Fam.	J/M	Mat.	Fam.	$\hbar\Omega_R$	$\hbar k/2$	$\hbar\gamma/2$	CR	S	V	sqrt (S/V)
D1	1	Kometani 2001	1	Ag-Au	Sphere	TC	Cy	J	BH^{4+}	Weak	High δ	-	-	-	-	-	-
D2	D1	Hranisavljievic 2002	3	Ag	Sphere	TC	Cy	J	PO^{4-}	Weak	High δ	-	-	-	-	-	-
D3	D2	Itoh 2003	59	Ag	Sphere	R6G	Rh	M	Other	Other	200	123	65	1.06	9.2	82.4	0.33
D4	D3	Liu 2007	60	Au	Sphere	Cc	P	M	Cisteine	Thiol	Double $\hbar\Omega_R$	-	-	-	-	-	-
D5	D3	Liu 2007	60	Ag	Sphere	Em	Po	M	Cisteine	Thiol	Double $\hbar\Omega_R$	-	-	-	-	-	-
D6	D4	Uwada 2007	61	Au	Sphere	PIC	Cy	J	MUA	Thiol	124	150	12.6	0.76	20.1	268.1	0.27
D7	5	Yoshida 2008	5	Ag	Sphere	TC	Cy	J	BH^{4+}	Weak	High δ	-	-	-	-	-	-
D8	11	Choi 2009	11	Au	Sphere	Cc	P	M	3-mercapto propionic acid	Thiol	112	149	30	0.63	7.9	65.4	0.35
D9	D5	Ni 2010	14	Au	Rods	HITC	Cc	M	PSS	Polymer	Double $\hbar\Omega_R$	-	-	-	-	-	-
D10	17	Balci 2013	17	Ag	Prism	TDBC	Cy	J	Citrate	Weak	204	310	15	0.63	No TEM		
D11	D6	Zengin 2013	62	Ag	Rods	TDBC	Cy	J	Citrate	Weak	100	250	50	0.33	12.7	100.3	0.36
D3	D7	Itoh 2014	63	Ag	Sphere	R6G	Rh	M	Not reported	-	200	114	65	1.12	5.0	33.5	0.39
D10	D8	Zengin 2015	64	Ag	Prism	TDBC	Cy	J	Citrate	Weak	290	92	15	2.71	6.3	21.2	0.55
D12	D9	Roller 2016	65	Au	Sphere	TDBC	Cy	J	DNA origami	Polymer	150	120	15	1.11	10.1	67.0	0.39
D13	27	Liu 2017	27	Ag-Au	Cubes	PIC	Cy	J	CTAB	Surfactant	220	89	12.5	2.17	10.7	81.2	0.36
D10	D10	Wersall 2017	66	Ag	Prism	TDBC	Cy	J	Citrate	Weak	400	75	15	4.44	2.7	7.6	0.60
D14	D11	Rodarte 2017	67	Ag	Sphere	AF488	Other	M	AUT	Thiol	603	112	83	3.09	17.7	220.9	0.28
D15	D11	Rodarte 2017	67	Ag	Sphere	AF488	Other	M	AHDT	Thiol	436	112	83	2.24	17.7	220.9	0.28
D16	D11	Rodarte 2017	67	Ag	Sphere	AF488	Other	M	APT	Thiol	366	112	83	1.88	17.7	220.9	0.28

Chronological				NPs		Dye			CL		Coupling parameters				Size parameters		
System #	Paper #	1st author & year	Ref.	Mat.	Shape	Name	Fam.	J/M	Mat.	Fam.	$\hbar\Omega_R$	$\hbar k/2$	$\hbar\gamma/2$	CR	S	V	sqrt (S/V)
D17	D11	Rodarte 2017	67	Ag	Sphere	AF488	Other	M	PEG-S	Thiol	125	112	83	0.64	17.7	220.9	0.28
D18	D12	Stete2017	68	Au	Sphere	TDBC	Cy	J	Tween20	Polymer	173	Not reported	-	-	-	-	-
D19	D12	Stete2017	68	Au	Rods	TDBC	Cy	J	Tween20	Polymer	144	Not reported	-	-	-	-	-
D20	D13	Stete2018	69	Au	Rods	TDBC	Cy	J	Citrate	Weak	232	160	23.5	1.26	0.8	1.5	0.71
D20	D13	Stete2018	69	Au	Rods	TDBC	Cy	J	Citrate	Weak	156	154.5	23.5	0.88	5.5	28.0	0.44
D20	D13	Stete2018	69	Au	Rods	TDBC	Cy	J	Citrate	Weak	145	155	23.5	0.81	10.9	84.2	0.36
D20	D13	Stete2018	69	Au	Rods	TDBC	Cy	J	Citrate	Weak	107	155	23.5	0.60	17.7	196.3	0.30
D21	33	Song 2019	33	Au	Cubes	PIC	Cy	J	Cl^-	Surfactant	130	277	12.6	0.45	43.4	614.1	0.27
D21	33	Song 2019	33	Au	Cubes	PIC	Cy	J	Cl^-	Surfactant	330	172	12.6	1.79	43.4	614.1	0.27
D10	D14	Wersall 2019	70	Ag	Prism	TDBC	Cy	J	Citrate	Weak	250	100	15	2.17	6.3	21.2	0.55
D22	40	Kumar 2020	40	Au	Rods	TDBC	Cy	J	CTAB	Surfactant	200	80	15	2.11	2.8	10.9	0.51
D22	40	Kumar 2020	40	Au	Rods	TDBC	Cy	J	CTAB	Surfactant	170	95	15	1.55	6.9	42.0	0.40
D22	40	Kumar 2020	40	Au	Rods	TDBC	Cy	J	CTAB	Surfactant	135	118	15	1.02	15.0	136.7	0.33
D23	42	Li 2020	42	Ag-Au	Cubes	TDBC	Cy	J	CPC	Surfactant	156	124	12.5	1.14	No TEM		
D24	42	Li 2020	42	Ag-Au	Rings	TDBC	Cy	J	CPC	Surfactant	200	111	12.5	1.63	No TEM		
D25	D15	Takeshima2020	71	Ag	Prism	TPP	P	M	Citrate	Weak	Double $\hbar\Omega_R$	-	-	-	-	-	-
D26	D16	Hasegawa2022	72	Au	Rods	TPPS	P	J	CTAB + NaOL	Surfactant	160	85	30	1.39	9.9	65.9	0.39

S2. Molecular nomenclature and abbreviations

Table S3. List of the molecular QEs with their full names and abbreviations.

QEs acronym	***QEs full name***
TC	*3,3'-Disulfopropyl-5,5'-dichlorothiacyanine sodium salt*
AF488	Alexa Fluor 488 tetrafluorophenyl
Cc	*Citocrome C*
CV	*Cresyl Violet 640*
Cy	*3,3'-diethylthiacyanine iodide*
Cy5	*1,3,3-trimethyl-2- [5-(1, 3, 3-trimethyl-1,3-dihydro-indol-2-ylidene)-penta-1,3- dienyl]-3H-indolium chloride*
Cy75	*5-Chloro-2-[3-[5-chloro-3-(4-sulfobutyl)-3H-benzothiazol-2-ylidene]-propenyl]-3-(4-sulfobutyl)-benzothiazol-3-ium hydroxide*
DBTC	*2,2'-dimethyl-8-phenyl-5,6,5',6'-dibenzothiacarbocyaninechloride*
DOTCI	*3,3' -diethyloxatricarbocyanine iodide*
Em	*Emoglobine*
FITC	*fluorescein isothiocyanate*
HITC	*1,1',3,3,3',3'-hexamethylindotricarbocyanine perchlorate*
IR806	*2-[2-[2-chloro-3-[2-[1,3-dihydro-3,3-dimethyl-1-(4-sulfobutyl)-2H-indol-2-ylidene]-ethylidene]-1-cyclopenten-1-yl]-ethenyl]-3,3-dimethyl-1-(4-sulfobutyl)-3H-indolium hydroxide, inner salt sodium salt*
JC1	*5,50,6,60-tetrachloro-1,10,3,30-tetraethyl-imidacarbocyanine iodide*
MB	*Methylene Blue*
MG	*Malachite green*
O720	*Oxazine 720*
O725	*Oxazine 725*
OC	*3,3'-disulfopropyl-5,5'- dichlorooxacyanine triethylammonium salt*
PIC	*1,1'-diethyl-2,2'-cyanine*
R640	*Rhodamine 640*
R6G	*Rhodamine 6G*
S2165	*2-[3-[1,1-dimethyl-3-(4- sulfobutyl)-1,3-dihydro-benzo[e]indol-2-ylidene]-propenyl]-1, 1-dimethyl-3-(4-sulfobutyl)-1H-benzo[e]indolium hydroxide*
S9	*2-([3-(2-[4-(Dimethylamino)phenyl]ethenyl)-5,5-dimethyl-2-cyclohexen-1-ylidene]methyl)-3-methylbenzothiazolium perchlorate, 2-[[3-[2-[4-(Dimethylamino)phenyl]ethenyl]-5,5-dimethyl-2-cyclohexen-1-ylidene]methyl]-3-methylbenzothiazolium perchlorate*
SO-PIC	*1,1'-sulphonatedipropyl-2,2'-cyanine*
SQ	*2,4-bis[(1-ethyl-3,3-dimethyl-2,3-dihydroindol-2-ylidene)- methyl]squaraine dye*
TC(Et)	*5,5'-dichloro-3,3'-disulfopropyl-9- ethylthiacarbocyanine triethylamine*
TC(Et)'	*5-Phenyl-2-[2-[[5-phenyl-3-(4- sulfobutyl)-3H-benzoxazol-2-ylidene]-methyl]-but-1-enyl]-3-(4-sulfobutyl)- benzoxazolium hydroxide*
TC(Ph)	*3,3'-disulfopropyl-5,5'-dichloro-9-phenylthiacarbocyanine triethylammonium salt*
TDBC	*5,5',6,6'-tetrachloro-1-1'-diethyl-3,3'-di(4-sulfobutyl)-benzimidazolocarbocyanine*
TPPS	Tetraphenyl porphyrin sulphonate

Table S4. List of the CL molecules with their full names and abbreviations.

CL molecules acronym	**CL molecules full name**
BH_4^+	BH_4^+
THCP	Tetrakis(hydroxymethyl)phosphonium chloride
3-mercaptopropionic acid	3-mercaptopropionic acid
AHDT	16-amino-1- hexadecanethiol hydrochloride
APT	6-amino-1-hexanethiol hydrochloride
AUT	11-amino- 1-undecanethiol hydrochloride
Cisteine	Cisteine
Citrate	Citrate
Cl^-	Cl^-
CPC	hexadecylpyridinium chloride monohydrate
CTAB	Hexadecyl trimethyl ammonium bromide
CTAC	Hexadecyl trimethyl ammonium chloride
DBAC	Benzyldimethylhexadecylammonium chloride
DNA origami	DNA origami
Mercaptoetansulphonate	Mercaptoetansulphonate
MUA	11-mercaptoundecanoic acid
PAH	Poly allylamine hydrochloride
PEG-S	Polyethilenglicole sulphur
PEI	Polyethileneimine
PSS	Sodium 4-styrenesulfonate
PVP	Polyvinilpirrolidone
PVSA	Polyvinylsulfonic acid
SOt	1-sulphonate octyl thiol
Thiocoline	Thiocoline
TMAOt	1-trimethyl ammonium octyl thiol
TMAUt	1-trimethyl ammonium undecyl thiol

S3. Additional notes about the definition of the dataset

S3.1 Retrieving optical and geometrical parameters.

The optical parameters $\hbar\Omega_R$, $\hbar\gamma$, $\hbar k$ were retrieved from the data reported in the original papers, if present. When these data were not directly available in the papers, we used the software https://apps.automeris.io/wpd/ to retrieve the plexcitonic peaks maxima in the extinction spectra and calculated $\hbar\Omega_R$ as the energy difference between them. When $\hbar k$ and $\hbar\gamma$ values were not reported, they were estimated as the full width at half maximum of the bands appearing in the extinction spectra.[73]

To estimate the effective volume V_{eff} of the NPs, we assumed that it could be approximated by their geometric volume V. For this reason, we calculated the V_{eff} from the geometrical dimensions of the

NPs extracted from the TEM analysis. The samples where the TEM analysis was not reported could not be included in the statistical analysis.

S3.2 Exclusions

In the analysis of $\hbar\Omega_R$ and CR reported in Figure 3, we had to exclude some samples of the dataset: (i) in the case of large detuning, $\hbar\Omega_R$ cannot be calculated simply as the difference between the energies of UP and LP, but more sophisticated fitting procedures are required, which require knowing the position of the polaritonic peaks for several detuning values.[74] When this information was not available, the samples were excluded from the analysis.[1,2,49,3–5,8,9,13,22,26]

(ii) in some works, one or both the plexcitonic bands were hidden by an excess of free dye in solution.[7,14,] In these cases, it is not trivial to reliably extract the ratio between the coupled and uncoupled dyes, leading to an over- or under-estimation which would generate a detrimental systematic error. This is also the case of ref [44], in which the authors proposed to use differential spectrophotometry to remove the contribution of the uncoupled molecules.

S4. Multiple linear regression

In multiple linear regression, a (dependent) response variable y is expressed as a function of (independent) predictor variables x_i by the model:

$$y = \beta_0 + \sum_i \beta_i \, x_i$$

where β_0 is the intercept and β_i are the regression coefficients.

To estimate the validity of the regression, different kinds of statistical indicators can be used:

- R-squared: percentage variation in the dependent variable explained by the independent variables.
- Adj. R-squared: R-squared adjusted for the number of variables in the regression.
- Prob(F-Statistic): quantifies the statistical significance of the regression model.
- p-value: quantifies the statistical significance of the regression coefficient.

Dummy Encoding

While the use of numerical variables in linear regression is straightforward, categorical variables must be converted using *dummy encoding*. Consider a categorical variable characterized by N categories. In dummy encoding, a dummy variable is created for each category of the categorical variable. These dummy variables are binary variables, taking values 1 or 0 depending on whether or not the sample

is part of that category. Since only $N-1$ dummy variables are linearly independent, one of them can be dropped in order to avoid multicollinearity.

Example: Consider the categorical variable Material. We have three samples (Sample A, Sample B and Sample C) characterized by nanoparticles made of different materials (Ag, Au, Ag/Au):

	Material
Sample A	Ag
Sample B	Au
Sample C	Ag/Au

By applying dummy encoding, a dummy variable is created for each category:

	Material Ag	Material Au	Material Ag/Au
Sample A	1	0	0
Sample B	0	1	0
Sample C	0	0	1

Since only two of these dummy variables are linearly independent, one out of three can be dropped, i.e., Material Ag/Au:

	Material Ag	Material Au
Sample A	1	0
Sample B	0	1
Sample C	0	0

S5. Surface area and volume for different NP shapes

The equations used to generate the curves in Figure 7d are the following, where l is the length variable that is varied, h is the height and r is the radius which are kept fixed.

	S	V	$\frac{S}{V}$
Sphere	$4\pi l^2$	$\frac{4}{3}\pi l^3$	$\frac{3}{l}$
Disk	$2\pi l(l+h)$	$\pi l^2 h$	$\frac{2(h+l)}{hl}$
Cube	$6l^2$	l^3	$\frac{6}{l}$
Rod	$2\pi r(r+l)$	$\pi r^2 l$	$\frac{2(r+l)}{rl}$

S6. Bivariate Plots

In the following figures, we report the scatter plots for CR vs $\sqrt{S/V}$ and Ω_R vs $\sqrt{S/V}$ for the various categorical variables (Material, Capping, Dye, Shape, Interaction, Aggregate) for the datasets, CPM-S + CPM-D, CPM-S, CPM-D.

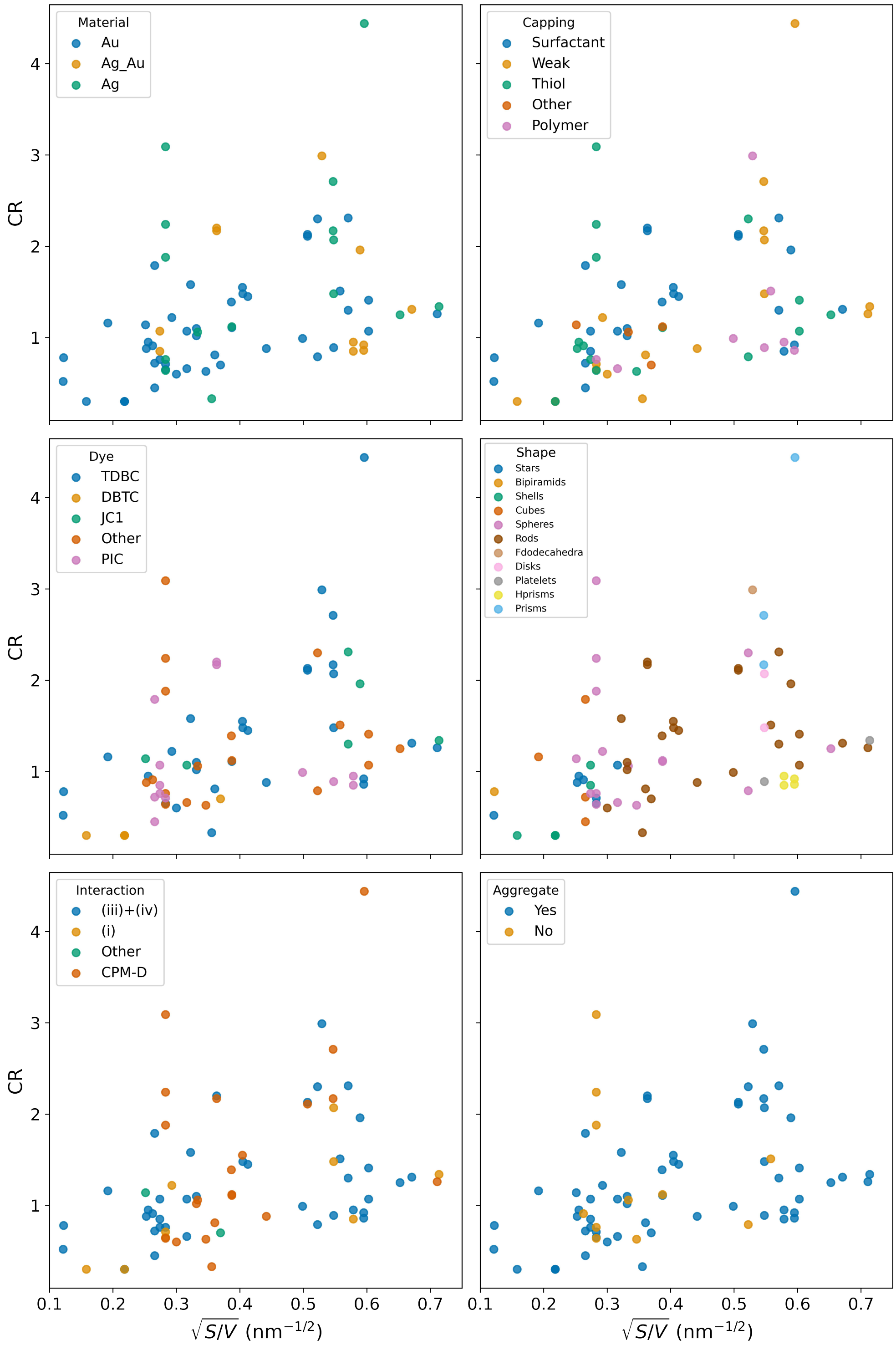

Figure S1. CR as a function of $\sqrt{S/V}$ for the various categorical variables (Dataset: CPM-S + CPM-D).

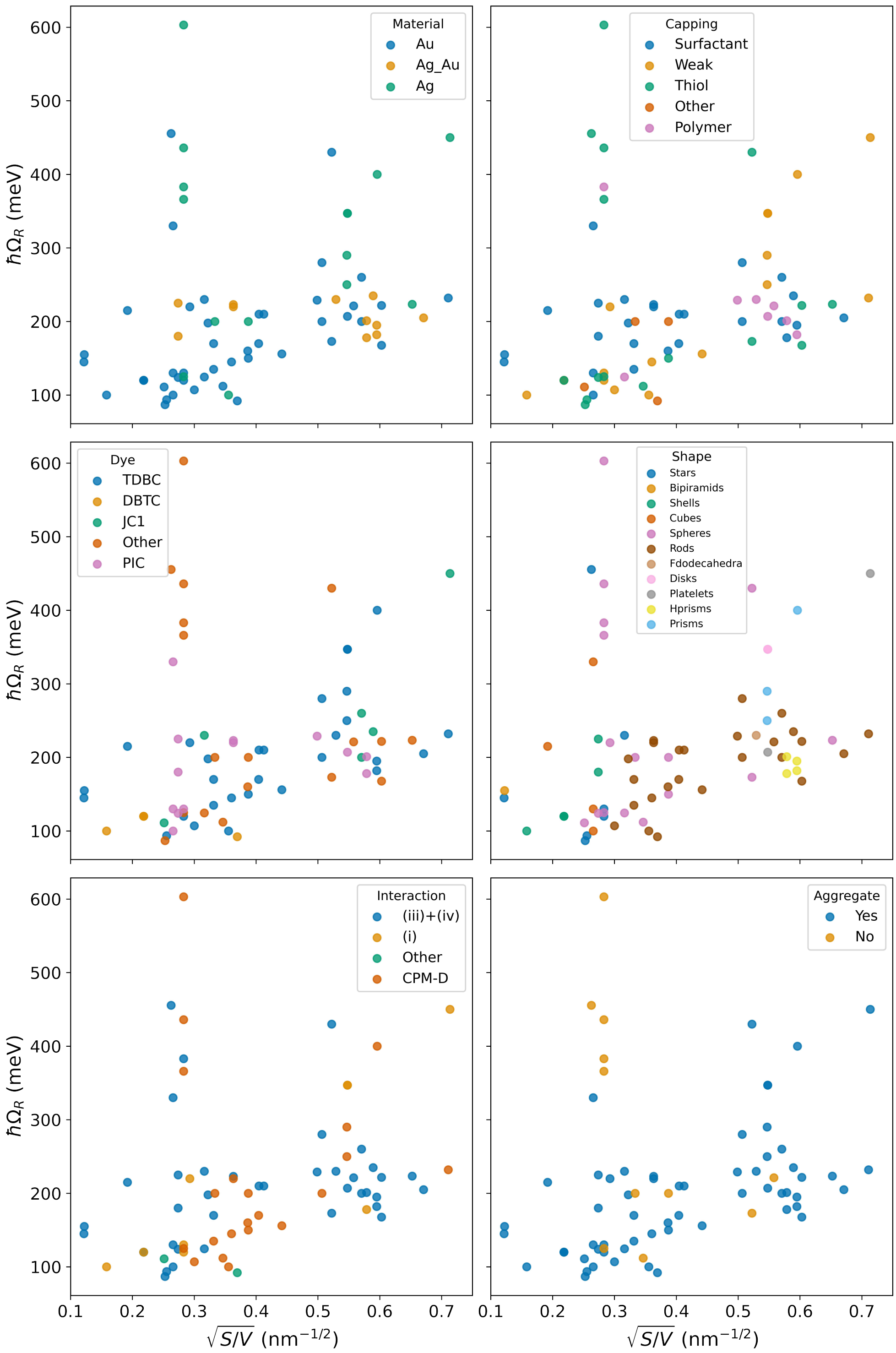

Figure S2. $\hbar\Omega_R$ as a function of $\sqrt{S/V}$ for the various categorical variables (Dataset: CPM-S + CPM-D).

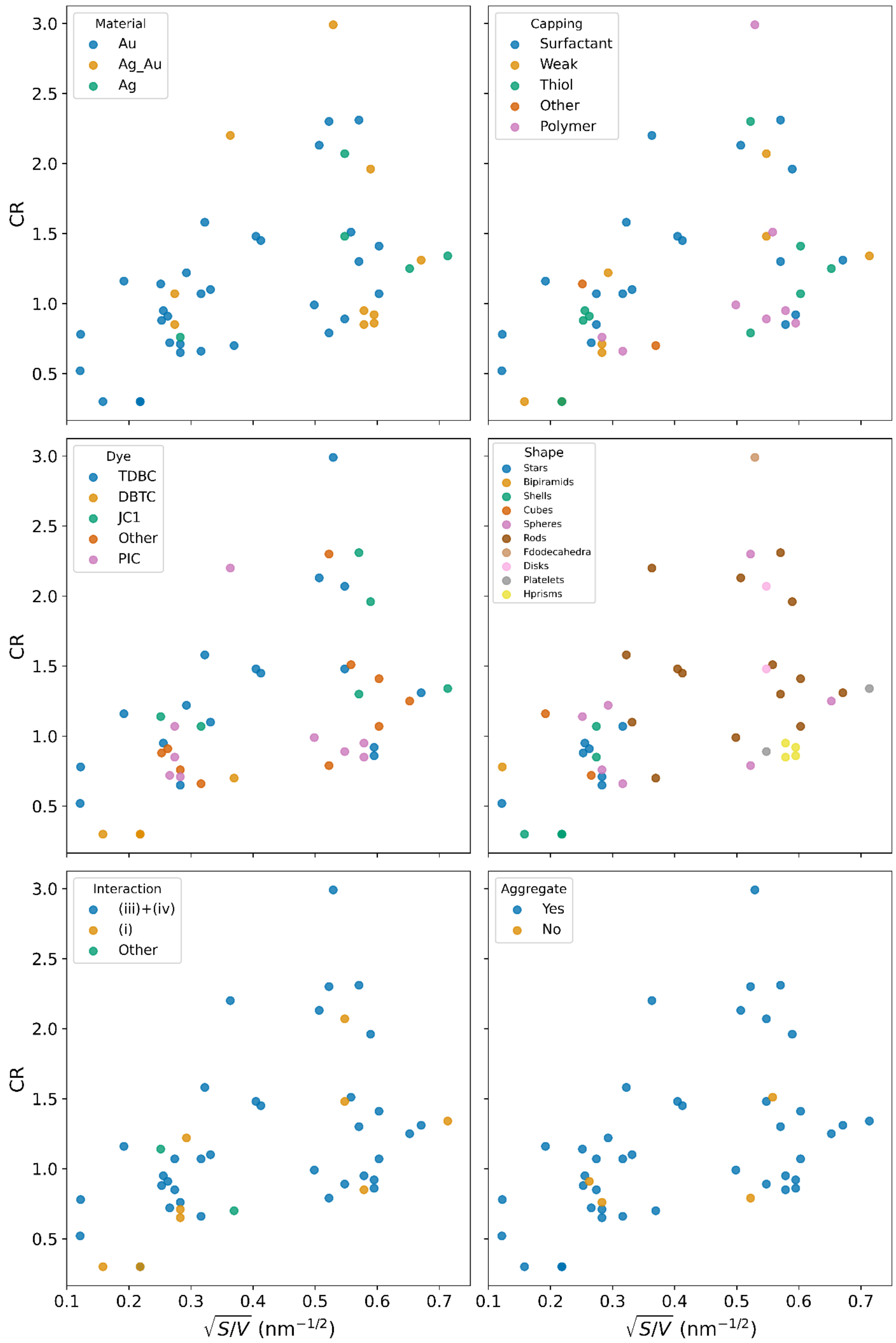

Figure S3. CR as a function of $\sqrt{S/V}$ for the various categorical variables (Dataset: CPM-S).

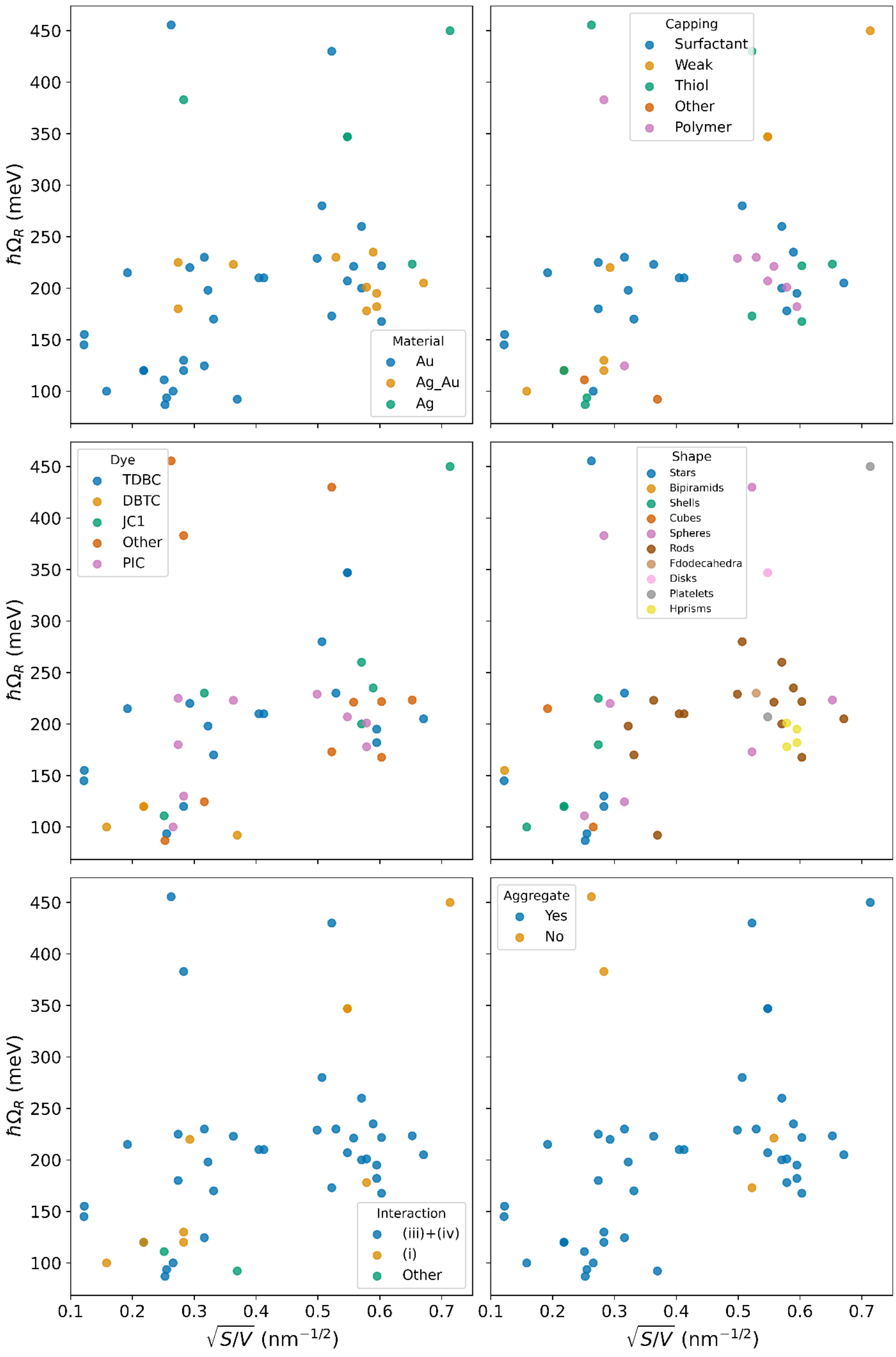

Figure S4. ħΩ_R as a function of $\sqrt{S/V}$ for the various categorical variables (Dataset: CPM-S).

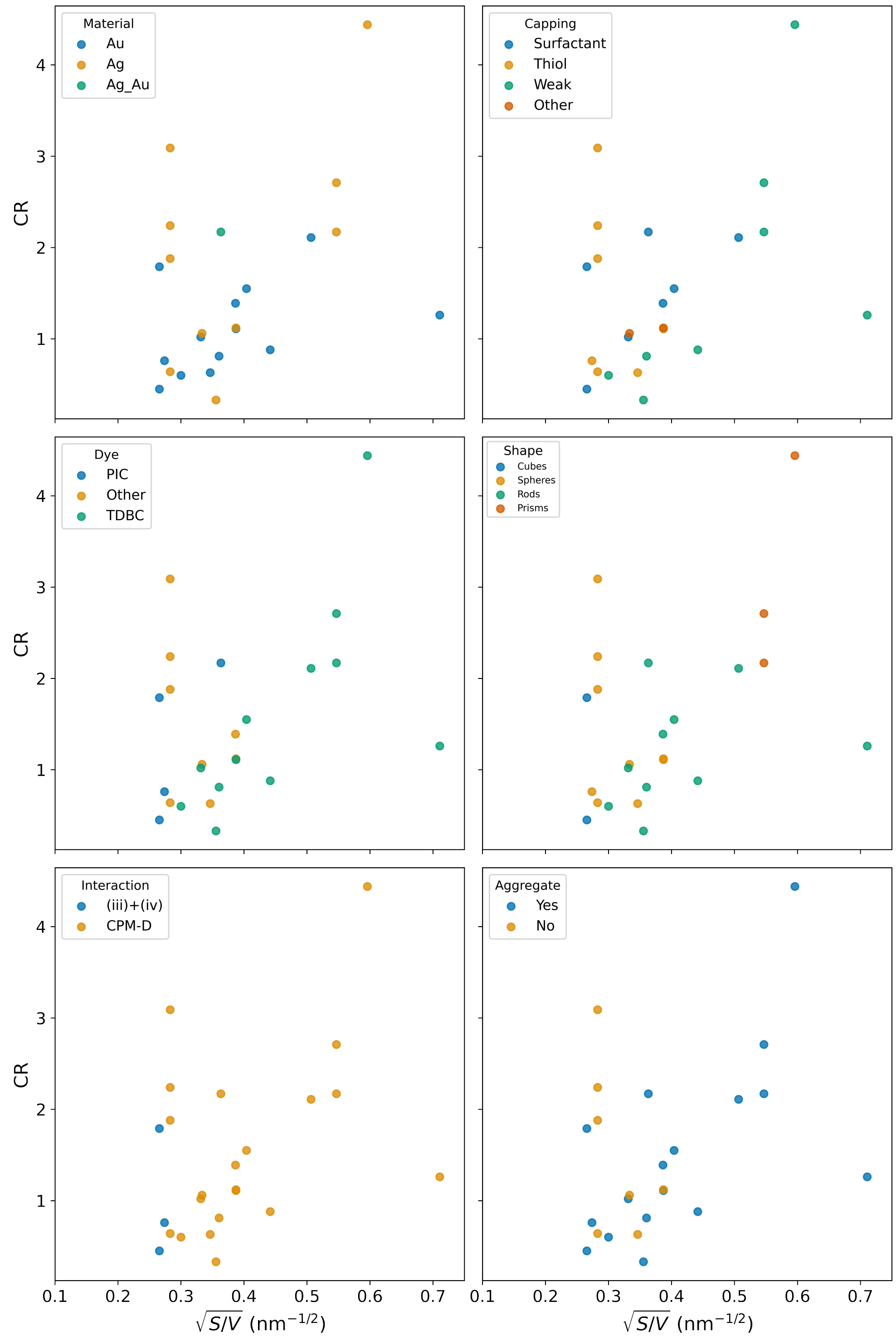

Figure S5. CR as a function of $\sqrt{S/V}$ for the various categorical variables (Dataset: CPM-D).

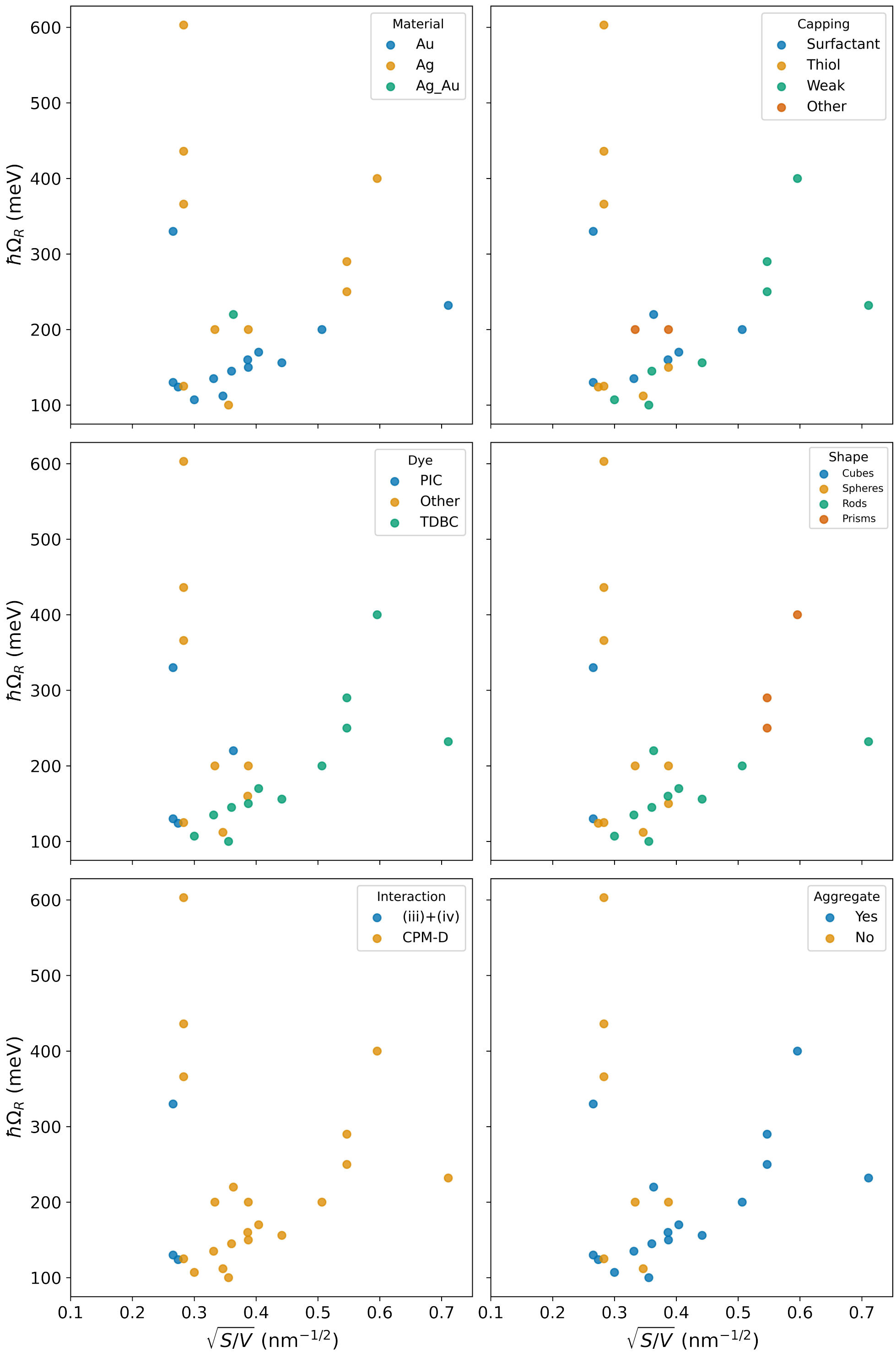

Figure S6. $\hbar\Omega_R$ as a function of $\sqrt{S/V}$ for the various categorical variables (Dataset: CPM-D).

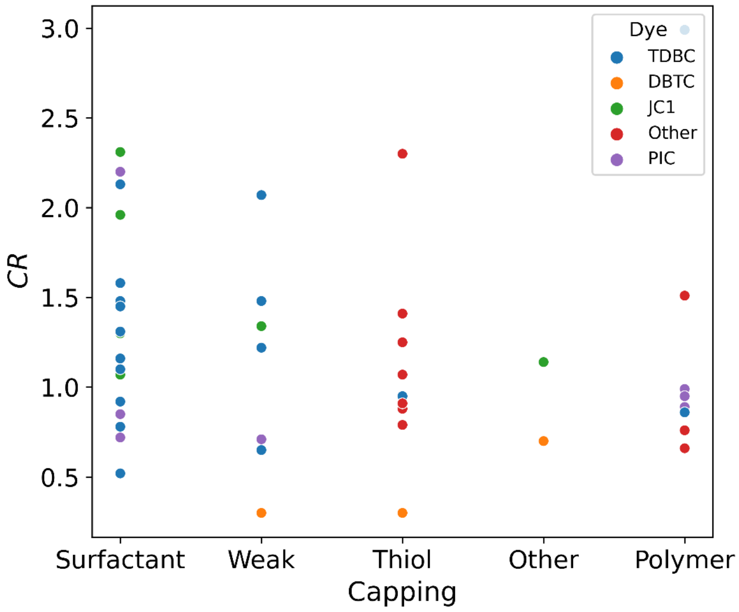

Figure S7. Multivariate plot of CR vs Capping Layer (x-axis) and Dye (colors) (Dataset:CPM-S).

S7. Linear Regression Models

The statistical analysis has been performed using the OLS (Ordinary Least Squares) class implemented in the Python library *Statsmodels*.[75] In the following are reported the output of the regression analysis, specifying the regression model (using R-style formulas) and the dataset used, either CPM-S + CPM-D, CPM-S, CPM-D.

OLS Model: CR ~ sqrt_S/V

Dataset: CPM-S + CPM-D

```
                            OLS Regression Results
==============================================================================
Dep. Variable:                     CR   R-squared:                       0.163
Model:                            OLS   Adj. R-squared:                  0.151
Method:                 Least Squares   F-statistic:                     13.44
Date:                Mon, 28 Nov 2022   Prob (F-statistic):           0.000479
Time:                        16:05:18   Log-Likelihood:                -73.550
No. Observations:                  71   AIC:                             151.1
Df Residuals:                      69   BIC:                             155.6
Df Model:                           1
Covariance Type:            nonrobust
==============================================================================
                 coef    std err          t      P>|t|      [0.025      0.975]
------------------------------------------------------------------------------
Intercept      0.4759      0.233      2.039      0.045       0.010       0.941
sqrt_S/V       2.0022      0.546      3.666      0.000       0.913       3.092
==============================================================================
Omnibus:                       30.238   Durbin-Watson:                   1.572
Prob(Omnibus):                  0.000   Jarque-Bera (JB):               55.711
Skew:                           1.548   Prob(JB):                     7.99e-13
Kurtosis:                       6.040   Cond. No.                         7.74
==============================================================================
```

OLS Model: Omega ~ sqrt_S/V

Dataset: CPM-S + CPM-D

```
                            OLS Regression Results
==============================================================================
Dep. Variable:                  Omega   R-squared:                       0.079
Model:                            OLS   Adj. R-squared:                  0.066
Method:                 Least Squares   F-statistic:                     5.927
Date:                Mon, 28 Nov 2022   Prob (F-statistic):             0.0175
Time:                        16:40:22   Log-Likelihood:                -426.48
No. Observations:                  71   AIC:                             857.0
Df Residuals:                      69   BIC:                             861.5
Df Model:                           1
Covariance Type:            nonrobust
==============================================================================
                 coef    std err          t      P>|t|      [0.025      0.975]
------------------------------------------------------------------------------
Intercept    135.4811     33.636      4.028      0.000      68.379     202.583
sqrt_S/V     191.6290     78.714      2.434      0.018      34.599     348.659
==============================================================================
Omnibus:                       38.486   Durbin-Watson:                   1.790
Prob(Omnibus):                  0.000   Jarque-Bera (JB):               85.581
Skew:                           1.892   Prob(JB):                     2.61e-19
Kurtosis:                       6.821   Cond. No.                         7.74
==============================================================================
```

OLS Model: CR ~ sqrt_S/V

Dataset: CPM-S

```
                            OLS Regression Results
==============================================================================
Dep. Variable:                     CR   R-squared:                       0.256
Model:                            OLS   Adj. R-squared:                  0.240
Method:                 Least Squares   F-statistic:                     15.50
Date:                Mon, 28 Nov 2022   Prob (F-statistic):           0.000284
Time:                        16:43:56   Log-Likelihood:                -34.152
No. Observations:                  47   AIC:                             72.30
Df Residuals:                      45   BIC:                             76.00
Df Model:                           1
Covariance Type:            nonrobust
==============================================================================
                 coef    std err          t      P>|t|      [0.025      0.975]
------------------------------------------------------------------------------
Intercept      0.4315      0.199      2.167      0.036       0.031       0.833
sqrt_S/V       1.7827      0.453      3.937      0.000       0.871       2.695
==============================================================================
Omnibus:                       11.006   Durbin-Watson:                   1.736
Prob(Omnibus):                  0.004   Jarque-Bera (JB):               10.677
Skew:                           1.073   Prob(JB):                      0.00480
Kurtosis:                       3.920   Cond. No.                         7.10
==============================================================================
```

OLS Model: Omega ~ sqrt_S/V

Dataset: CPM-S

```
                            OLS Regression Results
==============================================================================
Dep. Variable:                  Omega   R-squared:                       0.180
Model:                            OLS   Adj. R-squared:                  0.161
Method:                 Least Squares   F-statistic:                     9.855
Date:                Mon, 28 Nov 2022   Prob (F-statistic):            0.00299
Time:                        16:45:30   Log-Likelihood:                -273.57
No. Observations:                  47   AIC:                             551.1
Df Residuals:                      45   BIC:                             554.8
Df Model:                           1
Covariance Type:            nonrobust
==============================================================================
```

```
                 coef    std err          t      P>|t|      [0.025      0.975]
------------------------------------------------------------------------------
Intercept    112.3396     32.462      3.461      0.001      46.957     177.722
sqrt_S/V     231.7604     73.828      3.139      0.003      83.064     380.457
==============================================================================
Omnibus:                       22.269   Durbin-Watson:                   2.135
Prob(Omnibus):                  0.000   Jarque-Bera (JB):               31.471
Skew:                           1.606   Prob(JB):                     1.47e-07
Kurtosis:                       5.398   Cond. No.                         7.10
==============================================================================
```

OLS Model: CR ~ sqrt_S/V

Dataset: CPM-D

```
                            OLS Regression Results
==============================================================================
Dep. Variable:                     CR   R-squared:                       0.163
Model:                            OLS   Adj. R-squared:                  0.125
Method:                 Least Squares   F-statistic:                     4.287
Date:                Mon, 28 Nov 2022   Prob (F-statistic):             0.0504
Time:                        16:47:23   Log-Likelihood:                -30.687
No. Observations:                  24   AIC:                             65.37
Df Residuals:                      22   BIC:                             67.73
Df Model:                           1
Covariance Type:            nonrobust
==============================================================================
                 coef    std err          t      P>|t|      [0.025      0.975]
------------------------------------------------------------------------------
Intercept      0.2298      0.645      0.356      0.725      -1.108       1.567
sqrt_S/V       3.3218      1.604      2.070      0.050      -0.006       6.649
==============================================================================
Omnibus:                        5.459   Durbin-Watson:                   1.784
Prob(Omnibus):                  0.065   Jarque-Bera (JB):                3.807
Skew:                           0.955   Prob(JB):                        0.149
Kurtosis:                       3.401   Cond. No.                         9.96
==============================================================================
```

OLS Model: Omega ~ sqrt_S/V

Dataset: CPM-D

```
                            OLS Regression Results
==============================================================================
Dep. Variable:                  Omega   R-squared:                       0.003
Model:                            OLS   Adj. R-squared:                 -0.043
Method:                 Least Squares   F-statistic:                   0.05782
Date:                Mon, 28 Nov 2022   Prob (F-statistic):              0.812
Time:                        16:48:36   Log-Likelihood:                -149.39
No. Observations:                  24   AIC:                             302.8
Df Residuals:                      22   BIC:                             305.1
Df Model:                           1
Covariance Type:            nonrobust
==============================================================================
                 coef    std err          t      P>|t|      [0.025      0.975]
------------------------------------------------------------------------------
Intercept    201.6571     90.674      2.224      0.037      13.611     389.703
sqrt_S/V      54.2431    225.576      0.240      0.812    -413.572     522.059
==============================================================================
Omnibus:                       14.565   Durbin-Watson:                   1.387
Prob(Omnibus):                  0.001   Jarque-Bera (JB):               13.900
Skew:                           1.570   Prob(JB):                     0.000959
Kurtosis:                       5.009   Cond. No.                         9.96
==============================================================================
```

OLS Model: Omega ~ sqrt_S/V + Material_Ag + Material_Au

Dataset: CPM-S

```
                           OLS Regression Results
==============================================================================
Dep. Variable:                  Omega   R-squared:                       0.376
Model:                            OLS   Adj. R-squared:                  0.333
Method:                 Least Squares   F-statistic:                     8.646
Date:                Mon, 28 Nov 2022   Prob (F-statistic):           0.000132
Time:                        18:06:48   Log-Likelihood:                -267.13
No. Observations:                  47   AIC:                             542.3
Df Residuals:                      43   BIC:                             549.7
Df Model:                           3
Covariance Type:            nonrobust
===============================================================================
                  coef    std err          t      P>|t|      [0.025      0.975]
-------------------------------------------------------------------------------
Intercept     124.5901     44.400      2.806      0.008      35.050     214.131
sqrt_S/V      160.1020     74.607      2.146      0.038       9.643     310.561
Material_Ag   137.6136     40.874      3.367      0.002      55.184     220.043
Material_Au     3.4117     29.164      0.117      0.907     -55.402      62.226
==============================================================================
Omnibus:                       30.120   Durbin-Watson:                   2.314
Prob(Omnibus):                  0.000   Jarque-Bera (JB):               73.418
Skew:                           1.721   Prob(JB):                     1.14e-16
Kurtosis:                       8.064   Cond. No.                         10.3
==============================================================================
```

OLS Model: CR ~ sqrt_S/V + Capping_Surfactant

Dataset: CPM-S

```
                           OLS Regression Results
==============================================================================
Dep. Variable:                     CR   R-squared:                       0.315
Model:                            OLS   Adj. R-squared:                  0.283
Method:                 Least Squares   F-statistic:                     10.10
Date:                Mon, 28 Nov 2022   Prob (F-statistic):           0.000246
Time:                        17:17:04   Log-Likelihood:                -32.232
No. Observations:                  47   AIC:                             70.46
Df Residuals:                      44   BIC:                             76.01
Df Model:                           2
Covariance Type:            nonrobust
======================================================================================
                         coef    std err          t      P>|t|      [0.025      0.975]
--------------------------------------------------------------------------------------
Intercept              0.2915      0.206      1.413      0.165      -0.124       0.707
sqrt_S/V               1.8422      0.441      4.181      0.000       0.954       2.730
Capping_Surfactant     0.2863      0.148      1.935      0.059      -0.012       0.584
==============================================================================
Omnibus:                       16.764   Durbin-Watson:                   1.452
Prob(Omnibus):                  0.000   Jarque-Bera (JB):               20.873
Skew:                           1.251   Prob(JB):                     2.93e-05
Kurtosis:                       5.098   Cond. No.                         7.73
==============================================================================
```

OLS Model: CR ~ sqrt_S/V + Dye_TDBC_JC1:sqrt_S/V

Dataset: CPM-S

```
                           OLS Regression Results
==============================================================================
Dep. Variable:                     CR   R-squared:                       0.347
Model:                            OLS   Adj. R-squared:                  0.318
Method:                 Least Squares   F-statistic:                     11.70
Date:                Mon, 28 Nov 2022   Prob (F-statistic):           8.41e-05
Time:                        17:45:42   Log-Likelihood:                -31.085
No. Observations:                  47   AIC:                             68.17
Df Residuals:                      44   BIC:                             73.72
Df Model:                           2
Covariance Type:            nonrobust
=============================================================================================
                                coef    std err          t      P>|t|      [0.025      0.975]
```

```
------------------------------------------------------------------------------------------
Intercept                 0.4804      0.190      2.533      0.015       0.098       0.863
sqrt_S/V                  1.2552      0.479      2.621      0.012       0.290       2.220
Dye_TDBC_JC1:sqrt_S/V     0.8019      0.324      2.477      0.017       0.149       1.454
==============================================================================
Omnibus:                       12.716   Durbin-Watson:                   1.551
Prob(Omnibus):                  0.002   Jarque-Bera (JB):               13.431
Skew:                           1.067   Prob(JB):                      0.00121
Kurtosis:                       4.517   Cond. No.                         8.31
==============================================================================
```

OLS Model: CR ~ sqrt_S/V + CPM_CPM_D

Dataset: CPM-S + CPM-D

```
                            OLS Regression Results
==============================================================================
Dep. Variable:                     CR   R-squared:                       0.226
Model:                            OLS   Adj. R-squared:                  0.204
Method:                 Least Squares   F-statistic:                     9.955
Date:                Mon, 28 Nov 2022   Prob (F-statistic):           0.000161
Time:                        16:52:46   Log-Likelihood:                -70.752
No. Observations:                  71   AIC:                             147.5
Df Residuals:                      68   BIC:                             154.3
Df Model:                           2
Covariance Type:            nonrobust
================================================================================
                 coef    std err          t      P>|t|      [0.025      0.975]
--------------------------------------------------------------------------------
Intercept      0.3057      0.237      1.289      0.202      -0.168       0.779
sqrt_S/V       2.0914      0.530      3.945      0.000       1.033       3.149
CPM_CPM_D      0.3978      0.168      2.362      0.021       0.062       0.734
==============================================================================
Omnibus:                       20.892   Durbin-Watson:                   1.707
Prob(Omnibus):                  0.000   Jarque-Bera (JB):               29.327
Skew:                           1.205   Prob(JB):                     4.28e-07
Kurtosis:                       5.027   Cond. No.                         8.23
==============================================================================
```

S8. References

1. Kometani, N., Tsubonishi, M., Fujita, T., Asami, K. & Yonezawa, Y. Preparation and optical absorption spectra of dye-coated Au, Ag, and Au/Ag colloidal nanoparticles in aqueous solutions and in alternate assemblies. *Langmuir* **17**, 578–580 (2001).

2. Sato, T., Tsugawa, F., Tomita, T. & Kawasaki, M. Spectroscopic properties of noble metal nano-particles covered with J-aggregates of cyanine dye. *Chem. Lett.* **30**, 402–403 (2001).

3. Hranisavljevic, J., Dimitrijevic, N. M., Wurtz, G. A. & Wiederrecht, G. P. Photoinduced charge separation reactions of J-aggregates coated on silver nanoparticles. *J. Am. Chem. Soc.* **124**, 4536–4537 (2002).

4. Wiederrecht, G. P., Wurtz, G. A. & Hranisavljevic, J. Coherent coupling of molecular excitons to electronic polarizations of noble metal nanoparticles. *Nano Lett.* **4**, 2121–2125 (2004).

5. Yoshida, A., Kometani, N. & Yonezawa, Y. Silver:dye composite nanoparticles as a building unit of molecular architecture. *Colloids Surfaces A Physicochem. Eng. Asp.* **313–314**, 581–584 (2008).

6. Fofang, N. T. *et al.* Plexcitonic nanoparticles: Plasmon-Exciton Coupling in Nanoshell-J- Aggregate complexes. *Nano Lett.* **8**, 3481–3487 (2008).

7. Ni, W., Yang, Z., Chen, H., Li, L. & Wang, J. Coupling between molecular and plasmonic resonances in freestanding dye-gold nanorod hybrid nanostructures. *J. Am. Chem. Soc.* **130**, 6692–6693 (2008).

8. Yoshida, A., Yonezawa, Y. & Kometani, N. Tuning of the spectroscopic properties of composite nanoparticles by the insertion of a spacer layer: Effect of exciton-plasmon coupling. *Langmuir* **25**, 6683–6689 (2009).

9. Yoshida, A., Uchida, N. & Kometani, N. Synthesis and spectroscopic studies of composite gold nanorods with a double-shell structure composed of spacer and cyanine dye J-aggregate layers. *Langmuir* **25**, 11802–11807 (2009).

10. Juluri, B. K., Lu, M., Zheng, Y. B., Huang, T. J. & Jensen, L. Coupling between molecular and plasmonic resonances: Effect of molecular absorbance. *J. Phys. Chem. C* **113**, 18499–18503 (2009).

11. Choi, Y., Kang, T. & Lee, L. P. Plasmon resonance energy transfer (PRET)-based molecular imaging of cytochrome C in living cells. *Nano Lett.* **9**, 85–90 (2009).

12. Djoumessi Lekeufack, D. *et al.* Core-shell gold J-aggregate nanoparticles for highly efficient strong coupling applications. *Appl. Phys. Lett.* **96**, 1–3 (2010).

13. Yoshida, A. & Kometani, N. Effect of the interaction between molecular exciton and localized surface plasmon on the spectroscopic properties of silver nanoparticles coated with cyanine dye J-aggregates. *J. Phys. Chem. C* **114**, 2867–2872 (2010).

14. Ni, W. *et al.* Effects of dyes, gold nanocrystals, pH, and metal ions on plasmonic and molecular resonance coupling. *J. Am. Chem. Soc.* **132**, 4806–4814 (2010).

15. Hao, Y. W. *et al.* Hybrid-state dynamics of gold nanorods/dye J-aggregates under strong coupling. *Angew. Chemie - Int. Ed.* **50**, 7824–7828 (2011).

16. Fofang, N. T., Grady, N. K., Fan, Z., Govorov, A. O. & Halas, N. J. Plexciton dynamics: Exciton-plasmon coupling in a J-aggregate-Au nanoshell complex provides a mechanism for nonlinearity. *Nano Lett.* **11**, 1556–1560 (2011).

17. Balci, S. Ultrastrong plasmon–exciton coupling in metal nanoprisms with J-aggregates. *Opt. Lett.* **38**, 4498 (2013).

18. Melnikau, D., Savateeva, D., Susha, A., Rogach, A. L. & Rakovich, Y. P. Plasmon-exciton strong coupling in a hybrid system of gold nanostars and J-aggregates. *Nanoscale Res. Lett.* **8**, 2–7 (2013).

19. Balci, S. *et al.* Probing ultrafast energy transfer between excitons and plasmons in the ultrastrong coupling regime. *Appl. Phys. Lett.* **105**, 051105 (2014).

20. DeLacy, B. G. *et al.* Coherent Plasmon-Exciton Coupling in Silver Platelet-J-aggregate Nanocomposites. *Nano Lett.* **15**, 2588–2593 (2015).

21. Nan, F. *et al.* Unusual and Tunable One-Photon Nonlinearity in Gold-Dye Plexcitonic Fano Systems. *Nano Lett.* **15**, 2705–2710 (2015).

22. Fales, A. M., Norton, S. J., Crawford, B. M., DeLacy, B. G. & Vo-Dinh, T. Fano resonance in a gold nanosphere with a J-aggregate coating. *Phys. Chem. Chem. Phys.* **17**, 24931–24936 (2015).

23. Balci, S. *et al.* Tunable Plexcitonic Nanoparticles: A Model System for Studying Plasmon-Exciton Interaction from the Weak to the Ultrastrong Coupling Regime. *ACS Photonics* **3**, 2010–2016 (2016).

24. Melnikau, D. *et al.* Rabi Splitting in Photoluminescence Spectra of Hybrid Systems of Gold Nanorods and J-Aggregates. *J. Phys. Chem. Lett.* **7**, 354–362 (2016).

25. Simon, T. *et al.* Exploring the Optical Nonlinearities of Plasmon-Exciton Hybrid Resonances in Coupled Colloidal Nanostructures. *J. Phys. Chem. C* **120**, 12226–12233 (2016).

26. Hazra, B. *et al.* Hollow gold nanoprism as highly efficient 'Single' Nanotransducer for surface-enhanced raman scattering applications. *J. Phys. Chem. C* **120**, 25548–25556 (2016).

27. Liu, R. *et al.* Strong Light-Matter Interactions in Single Open Plasmonic Nanocavities at the Quantum Optics Limit. *Phys. Rev. Lett.* **118**, 1–6 (2017).

28. Das, K., Hazra, B. & Chandra, M. Exploring the coherent interaction in a hybrid system of hollow gold nanoprisms and cyanine dye J-aggregates: Role of plasmon-hybridization mediated local electric-field enhancement. *Phys. Chem. Chem. Phys.* **19**, 27997–28005 (2017).

29. Melnikau, D. *et al.* Strong Magneto-Optical Response of Nonmagnetic Organic Materials Coupled to Plasmonic Nanostructures. *Nano Lett.* **17**, 1808–1813 (2017).

30. Thomas, R. *et al.* Plexcitons: The Role of Oscillator Strengths and Spectral Widths in Determining Strong Coupling. *ACS Nano* **12**, 402–415 (2018).

31. Walters, C. M., Pao, C., Gagnon, B. P., Zamecnik, C. R. & Walker, G. C. Bright Surface-Enhanced Raman Scattering with Fluorescence Quenching from Silica Encapsulated J-Aggregate Coated Gold Nanoparticles. *Adv. Mater.* **30**, 1–6 (2018).

32. Kirschner, M. S. *et al.* Phonon-Driven Oscillatory Plasmonic Excitonic Nanomaterials. *Nano Lett.* **18**, 442–448 (2018).

33. Song, T. *et al.* Compounding plasmon-exciton strong coupling system with gold nanofilm to boost rabi splitting. *Nanomaterials* **9**, 1–10 (2019).

34. Melnikau, D. *et al.* Double Rabi Splitting in a Strongly Coupled System of Core-Shell Au@Ag Nanorods and J-Aggregates of Multiple Fluorophores. *J. Phys. Chem. Lett.* **10**, 6137–6143 (2019).

35. Kirschner, M. S. *et al.* Phonon-induced plasmon-exciton coupling changes probed via oscillation-associated spectra. *Appl. Phys. Lett.* **115**, 111903 (2019).

36. Balci, F. M., Sarisozen, S., Polat, N. & Balci, S. Colloidal nanodisk shaped plexcitonic nanoparticles with large rabi splitting energies. *J. Phys. Chem. C* **123**, 26571–26576 (2019).

37. Sun, L., Li, Z., He, J. & Wang, P. Strong coupling with directional absorption features of Ag@Au hollow nanoshell/J-aggregate heterostructures. *Nanophotonics* **8**, 1835–1845 (2019).

38. Guvenc, C. M., Balci, F. M., Sarisozen, S., Polat, N. & Balci, S. Colloidal Bimetallic Nanorings for Strong Plasmon Exciton Coupling. *J. Phys. Chem. C* **124**, 8334–8340 (2020).

39. Das, K., Dey, J., Verma, M. S., Kumar, M. & Chandra, M. Probing the role of oscillator strength and

charge of exciton forming molecular J-aggregates in controlling nanoscale plasmon-exciton interactions. *Phys. Chem. Chem. Phys.* **22**, 20499–20506 (2020).

40. Kumar, M., Dey, J., Verma, M. S. & Chandra, M. Nanoscale plasmon-exciton interaction: The role of radiation damping and mode-volume in determining coupling strength. *Nanoscale* **12**, 11612–11618 (2020).

41. Hendel, T., Krivenkov, V., Sánchez-Iglesias, A., Grzelczak, M. & Rakovich, Y. P. Strongly coupled exciton-plasmon nanohybrids reveal extraordinary resistance to harsh environmental stressors: Temperature, pH and irradiation. *Nanoscale* **12**, 16875–16883 (2020).

42. Li, N. *et al.* Strong plasmon-exciton coupling in bimetallic nanorings and nanocuboids. *J. Mater. Chem. C* **8**, 7672–7678 (2020).

43. Kirschner, M. S., Lin, X. M., Chen, L. X. & Schaller, R. D. Phase control of coherent acoustic phonons in gold bipyramids for optical memory and manipulating plasmon-exciton coupling. *Appl. Phys. Lett.* **116**, (2020).

44. Meera Mohankumar, Mahima Unnikrishnan, G. N. N. & Sanoop Mambully Somasundaran, Mavilakizhakke Puthiyaveetil Ajaykumar, Rotti Srinivasamurthy Swathi, and K. G. T. Finding the Needle in a Haystack: Capturing Veiled Plexcitonic Coupling through Differential Spectroscopy. *J. Phys. Chem. C* **124**, 26387–26395 (2020).

45. Stete, F., Bargheer, M. & Koopman, W. Optical non-linearities in plasmon-exciton core-shell particles: The role of heat. *arXiv:2009.00075 [physics.optics]* (2020).

46. Krivenkov, V., Samokhvalov, P., Nabiev, I. & Rakovich, Y. P. pH-Sensing Platform Based on Light-Matter Coupling in Colloidal Complexes of Silver Nanoplates and J-Aggregates. *J. Phys. Chem. C* **3**, 1972–1979 (2021).

47. Peruffo, N., Gil, G., Corni, S., Mancin, F. & Collini, E. Selective switching of multiple plexcitons in colloidal materials: directing the energy flow at the nanoscale. *Nanoscale* **13**, 6005–6015 (2021).

48. Peruffo, N., Mancin, F. & Collini, E. Plexcitonic Nanohybrids Based on Gold Nanourchins: The Role of the Capping Layer. *J. Phys. Chem. C* **125**, 19897–19905 (2021).

49. Finkelstein-Shapiro, D. *et al.* Understanding radiative transitions and relaxation pathways in plexcitons. *Chem* **7**, 1092–1107 (2021).

50. Guo, J. *et al.* Diverse axial chiral assemblies of J-aggregates in plexcitonic nanoparticles. *Nanoscale* **13**, 15812–15818 (2021).

51. Melnikau, D. *et al.* Strong coupling effects in a plexciton system of gold nanostars and J-aggregates. *J. Lumin.* **242**, 118557 (2021).

52. Balci, F. M. *et al.* Laser assisted synthesis of anisotropic metal nanocrystals and strong light-matter coupling in decahedral bimetallic nanocrystals. *Nanoscale Adv.* **3**, 1674–1681 (2021).

53. Zhu, J. *et al.* Strong Light-Matter Interactions in Chiral Plasmonic-Excitonic Systems Assembled on DNA Origami. *Nano Lett.* **21**, 3573–3580 (2021).

54. Wu, F. *et al.* Plexcitonic Optical Chirality: Strong Exciton-Plasmon Coupling in Chiral J-Aggregate-Metal Nanoparticle Complexes. *ACS Nano* **15**, 2292–2300 (2021).

55. Kumar, M., Dey, J., Swaminathan, S. & Chandra, M. Shape Dependency of the Plasmon-Exciton Interaction at the Nanoscale: Interplay between the Plasmon Local Density of States and the Plasmon Decay Rate. *J. Phys. Chem. C* **126**, 7941-7948 (2022).

56. Peruffo, N., Parolin, G., Collini, E., Corni, S. & Mancin, F. Engineering the aggregation of dyes on ligand-shell protected gold nanoparticles to promote plexcitons formation. *Nanomaterials* **12**, 1180 (2022).

57. Peruffo, N., Mancin, F. & Collini, E. Ultrafast dynamics of multiple plexcitons in colloidal nanomaterials. *J. Phys. Chem. Lett.* **13**, 6412-6419 (2022).

58. Melnikau, D. *et al.* Strong coupling effects in a plexciton system of gold nanostars and J-aggregates. *2Journal Lumin.* **242**, 1188557 (2022).

59. Itoh, T., Hashimoto, K., Ikehata, A. & Ozaki, Y. Direct demonstration for changes in surface plasmon resonance induced by surface-enhanced Raman scattering quenching of dye molecules adsorbed on single Ag nanoparticles. *Appl. Phys. Lett.* **83**, 5557–5559 (2003).

60. Liu, G. L., Long, Y. T., Choi, Y., Kang, T. & Lee, L. P. Quantized plasmon quenching dips nanospectroscopy via plasmon resonance energy transfer. *Nat. Methods* **4**, 1015–1017 (2007).

61. Uwada, T., Toyota, R., Masuhara, H. & Asahi, T. Single particle spectroscopic investigation on the interaction between exciton transition of cyanine dye J-aggregates and localized surface plasmon polarization of gold nanoparticles. *J. Phys. Chem. C* **111**, 1549–1552 (2007).

62. Zengin, G. *et al.* Approaching the strong coupling limit in single plasmonic nanorods interacting with J-aggregates. *Sci. Rep.* **3**, 1–8 (2013).

63. Itoh, T. *et al.* Single-molecular surface-enhanced resonance Raman scattering as a quantitative probe of local electromagnetic field: The case of strong coupling between plasmonic and excitonic resonance. *Phys. Rev. B - Condens. Matter Mater. Phys.* **89**, 1–8 (2014).

64. Zengin, G. *et al.* Realizing strong light-matter interactions between single-nanoparticle plasmons and molecular excitons at ambient conditions. *Phys. Rev. Lett.* **114**, 1–6 (2015).

65. Roller, E. M., Argyropoulos, C., Högele, A., Liedl, T. & Pilo-Pais, M. Plasmon-Exciton Coupling Using DNA Templates. *Nano Lett.* **16**, 5962–5966 (2016).

66. Wersall, M., Cuadra, J., Antosiewicz, T. J., Balci, S. & Shegai, T. Observation of mode splitting in photoluminescence of individual plasmonic nanoparticles strongly coupled to molecular excitons. *Nano Lett.* **17**, 551–558 (2017).

67. Rodarte, A. L. & Tao, A. R. Plasmon-Exciton Coupling between Metallic Nanoparticles and Dye Monomers. *J. Phys. Chem. C* **121**, 3496–3502 (2017).

68. Stete, F., Koopman, W. & Bargheer, M. Signatures of Strong Coupling on Nanoparticles: Revealing Absorption Anticrossing by Tuning the Dielectric Environment. *ACS Photonics* **4**, 1669–1676 (2017).

69. Stete, F., Schoßau, P., Bargheer, M. & Koopman, W. Size-Dependent Coupling of Hybrid Core-Shell Nanorods: Toward Single-Emitter Strong-Coupling. *J. Phys. Chem. C* **122**, 17976–17982 (2018).

70. Wersäll, M. *et al.* Correlative Dark-Field and Photoluminescence Spectroscopy of Individual Plasmon-Molecule Hybrid Nanostructures in a Strong Coupling Regime. *ACS Photonics* **6**, 2570–2576 (2019).

71. Takeshima, N. *et al.* Combined Use of Anisotropic Silver Nanoprisms with Different Aspect Ratios for Multi-Mode Plasmon-Exciton Coupling. *Nanoscale Res. Lett.* **15**, 15, (2020).

72. Hasegawa, S. & Imura, K. Photoluminescence Properties of Gold Nanorod and J-Aggregate Hybrid Systems Studied by Scanning Near-Field Optical Microscopy. *J. Phys. Chem. C* **126**, 5944–5949 (2022).

73. Pelton, M., Storm, S. D. & Leng, H. Strong coupling of emitters to single plasmonic nanoparticles: Exciton-induced transparency and Rabi splitting. *Nanoscale* **11**, 14540–14552 (2019).

74. Hertzog, M., Wang, M., Mony, J. & Börjesson, K. Strong light-matter interactions: A new direction within chemistry. *Chem. Soc. Rev.* **48**, 937–961 (2019).

75. Seabold, S. & Perktold, J. Statsmodels: Econometric and Statistical Modeling with Python. *Proc. 9th Python Sci. Conf.* 92–96 (2010) doi:10.25080/majora-92bf1922-011.
